# Metabolomic profiling of *Burkholderia thailandensis* infection of airway epithelial cells provides insights into potential therapeutic targets

**DOI:** 10.1128/msystems.00611-25

**Published:** 2025-10-31

**Authors:** Daniel J. Hicks, Nicole Aiosa, Anupama Sinha, Olakunle A. Jaiyesimi, Steven S. Branda, Neha Garg

**Affiliations:** 1 School of Chemistry and Biochemistry, Georgia Institute of Technology 1372, Atlanta, Georgia, USA; 2 Biotechnology & Bioengineering, Sandia National Laboratories 1105, Livermore, California, USA; 3 Center for Microbial Dynamics and Infection, Georgia Institute of Technology 1372, Atlanta, Georgia, USA; DOE Joint Genome Institute, Berkeley, California, USA

**Keywords:** infection culture metabolomics, melioidosis, *Burkholderia*, airway epithelial cells, HILIC

## Abstract

*Burkholderia pseudomallei,* a gram-negative saprophyte that causes melioidosis, has been classified as a potential bioweapon, posing a serious global threat. Metabolic re-wiring, production of virulence effectors, evasion/suppression of host defenses, and modification of host cell functions constitute important mechanisms in the pathogenesis of infection. Elucidating the metabolism during intracellular growth of the pathogen is critical for learning the mechanisms by which infection is accomplished. Currently, the metabolic activities associated with, and potentially mediating, host-pathogen interactions during infection are not well understood, but recent advances in untargeted mass spectrometry-based metabolomics methods are enabling the narrowing of this knowledge gap. Here, we used untargeted metabolomics analysis to identify polar metabolites produced by *Burkholderia thailandensis* E264 (a surrogate for *B. pseudomallei*) and airway epithelial cells (the murine cell line LA-4) during the intracellular stage of infection. Pathway analysis of annotated metabolites that differed in abundance in mock-challenged versus *B. thailandensis*-challenged host cells revealed changes in the activity of a diverse set of metabolic pathways that could be targeted to combat *Burkholderia* infections. These include pathways that mediate metabolic processes occurring in both the pathogen and host (e.g., polyamine biosynthesis, NAD^+^ [nicotinamide adenine dinucleotide] metabolism, and the citric acid cycle), as well as several pathogen-specific pathways (peptidoglycan biosynthesis, ornithine lipid production, and quorum sensing-regulated secondary metabolites). The observed shift in the metabolome shows commonalities with other gram-negative pathogens during infection. Our results provide insight into the changes in metabolism associated with *Burkholderia* infection and reveal several promising targets for therapeutic interventions.

**IMPORTANCE:**

*Burkholderia pseudomallei* is the causative agent of infectious disease, namely melioidosis. When inhaled, *Burkholderia pseudomallei* causes severe respiratory infections. Due to the potential for severe airborne infections, it is classified as a Tier 1 biothreat agent. The intrinsic antibiotic resistance and increased global prevalence necessitate the development of alternative treatments. Infection triggers a metabolic “arms race” between host and pathogen, where both organisms dramatically alter their metabolism to outcompete one another. By studying these changes, one can identify new therapeutic targets for drug discovery and better understand the mechanisms pathogens use to establish and maintain infection. We performed an untargeted metabolomics analysis of murine epithelial cells co-cultured with *Burkholderia thailandensis*, a surrogate for *Burkholderia pseudomallei*, to identify the metabolic shifts that occur during intracellular infection. Using these analyses, we propose several pathways and therapeutic interventions to enable pathogen clearance.

## INTRODUCTION

*Burkholderia pseudomallei* (*Bp*) is a gram-negative, soil-dwelling bacterial pathogen that is the etiological agent for the human disease melioidosis. Due to the absence of an FDA-approved vaccine against melioidosis, as well as the intrinsic multi-drug resistance of *Bp*, there continue to be many infections with high morbidity and mortality in the tropics and sub-tropics (1–4). The challenge posed by *Bp* is further amplified by its multiple routes of entry (inhalation, ingestion, and skin abrasion), as well as its diverse clinical presentations (5, 6). In the last 3 years, there has been an increase in the prevalence of *Bp* in the continental USA, including an outbreak of non-travel-related melioidosis across several states (tracing back to contaminated consumer products) and isolation of *Bp* from the soil in the Gulf Coast region of Mississippi, establishing endemic status in the region (7–9). Thus, *Bp* is a rising concern for both public health and national security, which calls for the development of alternatives to the currently inadequate prevention and treatment methods (10, 11).

The varied symptoms and intra-host evolution that are hallmarks of melioidosis (12, 13) demand a creative approach to investigating alternative countermeasures. To this end, metabolomic profiling can provide valuable insight into the chemistry of host-pathogen interactions, thereby informing the development of new countermeasures. Because an organism’s metabolome is often highly dependent on context, metabolomics is a powerful tool to gain insight into both global and pathway-specific responses to different stimuli in biological systems. Targeted metabolomics methods are limited to the investigation of one or a few predefined biochemical pathways, whereas untargeted metabolomics methods enable simultaneous analysis of a multitude of pathways without the need for prior knowledge of their relevance. Metabolomics analysis of cultures, in which the pathogen and host cell interact through co-culture *in vitro*, can be a powerful means by which to characterize the chemical changes in both pathogen and host during infection, to support hypothesis generation as well as functional analysis of pathway activity.

A mammalian cell culture-based approach offers several advantages over animal models, including lower cost, greater experimental control, and fewer ethical considerations. Thus, whenever possible, biochemical insights can be discovered via metabolome profiling of mammalian cells and then validated in animal models. In animals, metabolites are frequently transported between organs and tissues. Thus, the overall changes in metabolites can be very different from the responses of specific cellular phenotypes (14). In addition, whole tissue homogenates commonly used in metabolomics studies contain many different cell types. Lastly, a metabolite can arise from inputs from multiple metabolic pathways and serve diverse functions. Thus, analysis of different pathways that feed into the biosynthesis or catabolism of a given metabolite should be carefully considered. In this study, we attempt such an analysis of untargeted metabolomics of airway epithelial cells (AECs).

We previously established infection culture models for *Burkholderia thailandensis* (*Bt*) and host cells for the purpose of characterizing their chemical interactions during infection (15). *Bt* is a close relative of *Bp* that shares pathogenic mechanisms and behaviors in cultured cells and animal models, but is only mildly pathogenic to immunocompetent humans, and therefore is commonly used as a safe surrogate for *Bp* (16–18). Previous studies by our group and others had indicated that *Bt* or *Bp* infection of host cells typically causes significant changes in gene and protein expression, including signature alterations associated with exposure to the intracellular environment (19–23). Our initial analysis of *Bt* infection cultures revealed stark differences in the metabolomes of both pathogen and host depending upon the multiplicity of infection (MOI) (the number of bacteria relative to the number of host cells, i.e., dosage) and the stage of infection (in particular, immediately following internalization [3 h post-exposure; 3 hpe] versus after prolonged intracellular interaction [24 hpe]) (15). While this effort succeeded in providing new insights into *Burkholderia* pathogenesis and host defense responses, the methods utilized for metabolite extraction and chromatographic separation imposed a bias toward analysis of non-polar metabolites such that they did not examine the possibility of infection-associated changes in polar metabolites. A potentially important source of polar metabolites during infection is central metabolism in the host cell. These polar metabolites (referred to as the primary metabolome) are known to play key roles in innate and adaptive immune responses, stress responses, and maintenance of homeostasis (24, 25). Moreover, the interface between central metabolism and the immune system is thought to evolve over the course of infection, such that polar metabolites associated with this interface may serve as contextual cues for pathogens to regulate expression of their virulence factors (26, 27). Thus, we sought to characterize the chemical interactions between *Burkholderia* and host cells with respect to polar metabolites, placing particular emphasis on investigating how the pathogen utilizes the host’s central metabolism and adapts its own metabolism to enhance intracellular survival and pathogenesis. To this end, infection cultures comprised of *Bt* and AECs (i.e., *Bt*:AEC co-cultures) were analyzed using hydrophilic liquid chromatography (HILIC) mass spectrometry-based metabolomics, which enabled identification of infection-associated changes in polar metabolite profiles. We matched the metabolites of interest to their respective metabolic pathways and compared the indicated patterns of pathway activity to those reported in other studies of infection. In many cases, this approach enabled identification of the driver (pathogen versus host) for the observed metabolic changes, the pathways supporting *Bt* pathogenesis and intracellular survival, and the pathway components that constitute the most promising targets for therapeutic intervention. In summary, our analysis of polar metabolites produced during *Bt* infection of AECs has provided new insight into the chemical features of the host-pathogen interaction, revealed metabolic shifts and immunometabolic cross-talk that likely hold important implications for the ultimate fate of pathogen and host, and enabled identification of opportunities to exploit apparent vulnerabilities in the pathogen’s metabolism and thereby favor host success in safely resolving *Burkholderia* infections.

## RESULTS AND DISCUSSION

### Metabolomic profiling of *B. thailandensis* and airway epithelial cells in co-culture

**Fig 1:**
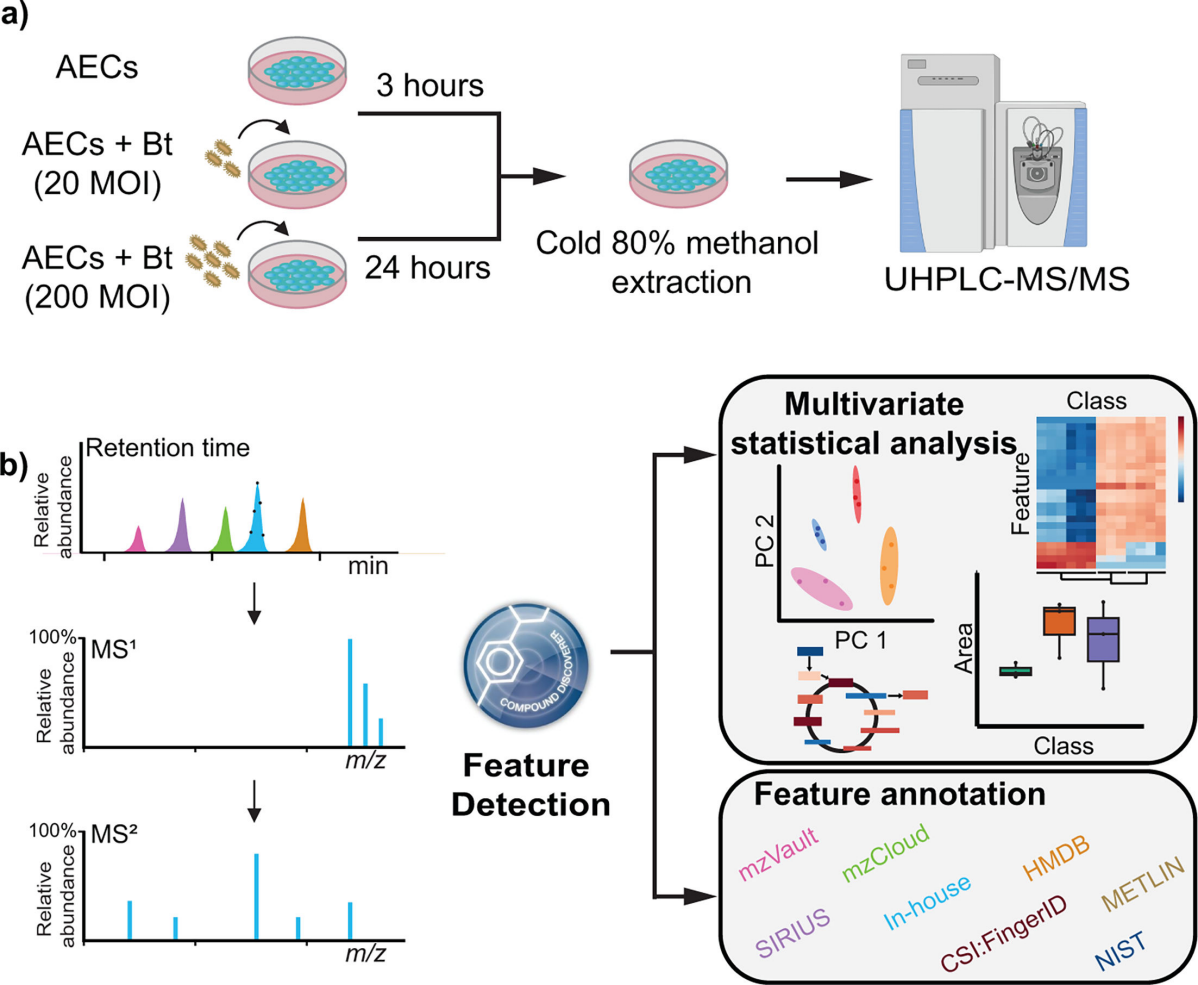
Overview of study design, sample collection and processing, and metabolomics data acquisition and analysis. (**a**) Illustration of the experimental workflow. AECs were challenged with *Bt* at an MOI of 20 or 200; the AECs (including internalized *Bt*) were collected at 3 or 24 hpe and extracted with cold 80% methanol; and metabolomics data were acquired via UHPLC-MS/MS. (**b**) Illustration of the metabolomics data analysis workflow and tools.

The airway epithelium is the first line of defense against *Burkholderia* and other respiratory pathogens, acting as a key initiator and orchestrator of host defense responses (28, 29). Recent studies have also linked atypical central metabolism in AECs to several chronic respiratory diseases (30). Leveraging our previously established AEC model of *Burkholderia* infection (15), the murine AEC line LA-4 (31, 32) was challenged with *B. thailandensis* E264 (*Bt*) at an MOI of either 20 or 200; the co-cultures were collected at 3 and 24 hpe; the AECs (including intracellular *Bt*) were recovered and extracted with cold 80% methanol; and the extracts were subjected to HILIC separation for untargeted ultra-high-performance liquid chromatography-tandem mass spectrometry (UHPLC-MS/MS) data acquisition in positive and negative mode (Fig. 1).

**Fig 2:**
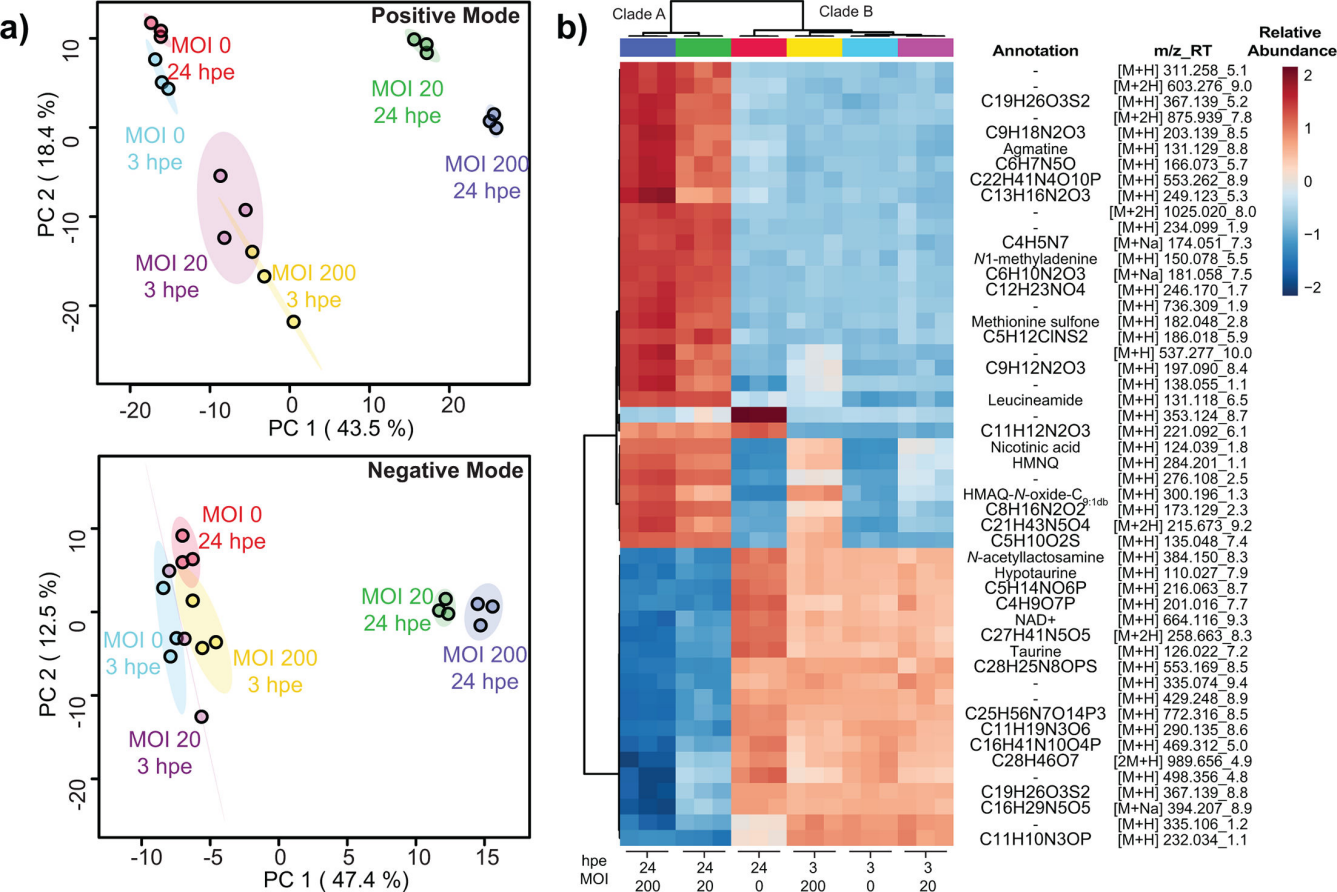
Multivariate analysis. (**a**) PCA score plots showing the metabolomic profiles of mock-challenged versus *Bt*-challenged AECs as assessed through data acquisition in positive and negative mode. (**b**) Heatmap of the top 50 features for HCA of positive-mode data. The relative abundance of each feature, along with its annotation or proposed chemical formula (where possible), is shown.

Principal component analysis (PCA) was performed to visualize the relationships between global metabolomic profiles of mock-challenged (MOI 0) and *Bt*-challenged (MOI 20 and 200) AECs collected at 3 and 24 hpe. The first and second components of the PCA models together captured 61.9% and 59.9% of the total variation in metabolomic profiles observed through analysis of the positive- and negative-mode data sets, respectively (Fig. 2a). In general, extracts derived from replicate cultures showed similar metabolomic profiles, as evident from the proximity of replicates in PCA space; this indicates low variability between these replicates in the experiment. Separation across principal component 1 (PC1) correlated well with differences in collection time: PC1 scores were generally negative for extracts collected at 3 hpe and positive for those collected at 24 hpe, with the notable exception of mock-challenged AECs (MOI 0) collected at 24 hpe. Separation across principal component 2 (PC2) roughly correlated with differences in MOI, such that lower PC2 scores were typically associated with higher MOIs. Separation across PC1 and PC2 in combination (PC1•PC2) revealed several striking trends. For instance, the extracts derived from mock-challenged AECs (MOI 0) showed similar PC1•PC2 scores regardless of collection time (3 versus 24 hpe), indicating that in the absence of *Bt*, the metabolomic profile of AECs generally remained stable for the duration of the experiment. In contrast, extracts derived from *Bt*-challenged AECs clustered primarily by collection time regardless of MOI (20 versus 200), indicating that in *Bt*-AEC co-cultures, it is the duration of the host-pathogen interaction and the responses that it elicits, rather than the pathogen dose, that most affects the global metabolic profile. Additionally, in the positive-mode data set, the extracts derived from mock-challenged AECs (MOI 0) were well separated from those derived from *Bt*-challenged AECs collected at 3 hpe, indicating that even a brief period of co-culture (or perhaps the mere presence of *Bt* itself) was sufficient to substantially change the global metabolic profile. For the most part, the trends observed here (polar metabolites) closely resembled those observed in our previous study (non-polar metabolites) (15), with the exception that differences in MOI were found to have less impact on global polar metabolomic profiles than on global non-polar metabolomic profiles. In summary, PCA revealed clear differences in the global metabolomic profiles of mock-challenged versus *Bt*-challenged AECs, and that the differences in global polar metabolomic profiles became more prominent as infection progressed. In concurrence with the PCA clustering, hierarchical clustering analysis (HCA) of each data set revealed that the culture conditions clustered into two major clades, with clade A comprised of *Bt-*challenged AECs collected at 24 hpe, and clade B comprised of all other culture conditions (mock- and *Bt*-challenged AECs collected at 3 hpe, plus mock-challenged AECs collected at 24 hpe) (Fig. 2b). This distinct clustering pattern further supports the conclusion that *Bt* challenge was associated with profound changes to the polar metabolome that became much more evident as the infection progressed from 3 hpe to 24 hpe.

To identify metabolite features associated with the observed profile shifts, HCA of the top 50 features was performed; the results are shown in Fig. 2b (positive-mode data set) and Fig. S2 (negative-mode data set). Features identified by HCA were annotated by searching spectral libraries and comparison with analytical standards (Table 1). For annotated metabolites, pathway analysis was performed to identify significantly altered metabolic pathways and cellular processes. This process revealed significant shifts in several key pathways in central metabolism, including the polyamine pathway, the NAD^+^ (nicotinamide adenine dinucleotide) salvage pathway, and the tricarboxylic acid (TCA) cycle, as described below. While much of central metabolism is conserved across species, subtle differences in biochemical pathways can result in complex immunometabolic cross-talk during co-culture (33, 34). Determining the source (bacterial vs host) of these differences can be challenging, as many biochemical pathways are shared between the pathogen and host; for instance, metabolites derived from tryptophan, such as serotonin, can be produced both by gram-negative bacteria and mammalian cells. Thus, analysis of these pathways requires disentanglement of bacteria-derived metabolites from host-derived metabolites. In contrast, specialized metabolites such as quorum sensing signals are produced only by bacteria, and the significance of their production can be readily gleaned. Thus, a primary objective of this study was to uncover the host-pathogen cross-talk in central metabolism to identify potential avenues for treatment interventions, both pathogen-directed (to disrupt bacterial processes that support infection) and host-directed (to enhance defenses and mitigate unproductive responses).

**TABLE 1:** Overview of metabolites in altered pathways^*a*^

Metabolite	Pathway	Annotation level (35)
Arginine	Polyamine	2
Agmatine [**↑**]		2
Ornithine [↓]		2
Putrescine [**↑**]		1
*N*-acetylputrescine [**↑**]		2
Diacetylputrescine [**↑**]		1
Methylthioadenosine [↓]		2
*S*-adenosylmethionine [↓]		1
Spermidine [↓]		2
Spermine [↓]		2
*N*-acetylspermine [↓]		2
Nicotinic acid [**↑**]	NAD^+^ metabolism	2
NAD^+^ [↓]		2
Nicotinamide [↓]		1
Nicotinamide riboside [↓]		3
Methylnicotinamide [↓]		2
Citric acid [↓]	TCA cycle	2
Pyruvic acid		2
Itaconic acid [↓]		2
Glutamic acid [↓]		2
Glutamine [↓]		2
Fumaric acid [↓]		2
Malic acid [↓]		2
*N1*-methyladenine [**↑**]	Nucleotide metabolism	1
*N3*-methyladenine [**↑**]		1
*N6*-methyladenine [**↑**]		1
*N7*-methylguanine [**↑**]		1
5-Hydroxy-methylcytosine [**↑**]		2 (36)
Adenine [↓]		2
UDP-GlcNAc [↓]	Peptidoglycan and sugar metabolism	2
UDP-MurNAc [**↑**]		3
UDP-MurNAc-pentapeptide [**↑**]		3
Alanine [↓]		2
Alanyl-alanine [**↑**]		2
LacNAc [↓]		2
UDP-Gal [↓]		2
Tryptophan [↓]	Tryptophan metabolism	2
Kynurenine		2
Kynurenic acid		2
Indolepyruvic acid [↓]		2
Indolelactic acid [**↑**]		2
Taurine [↓]	Taurine metabolism	1
Hypotaurine [↓]		2
Oxidized glutathione [↓]	Glutathione metabolism	2
Glutathione [↓]		2
Ophthalmic acid [↓]		2
Urocanic acid [**↑**]	Histidine degradation	2

^*a*^ [↑] indicates elevation and [↓] indicates depletion of the metabolite in the 200 MOI, 24 hpe condition compared to the 0 MOI, 24 hpe condition. Annotation level determined based on the Schymanski scale.

### Polyamine pathway

**Fig 3:**
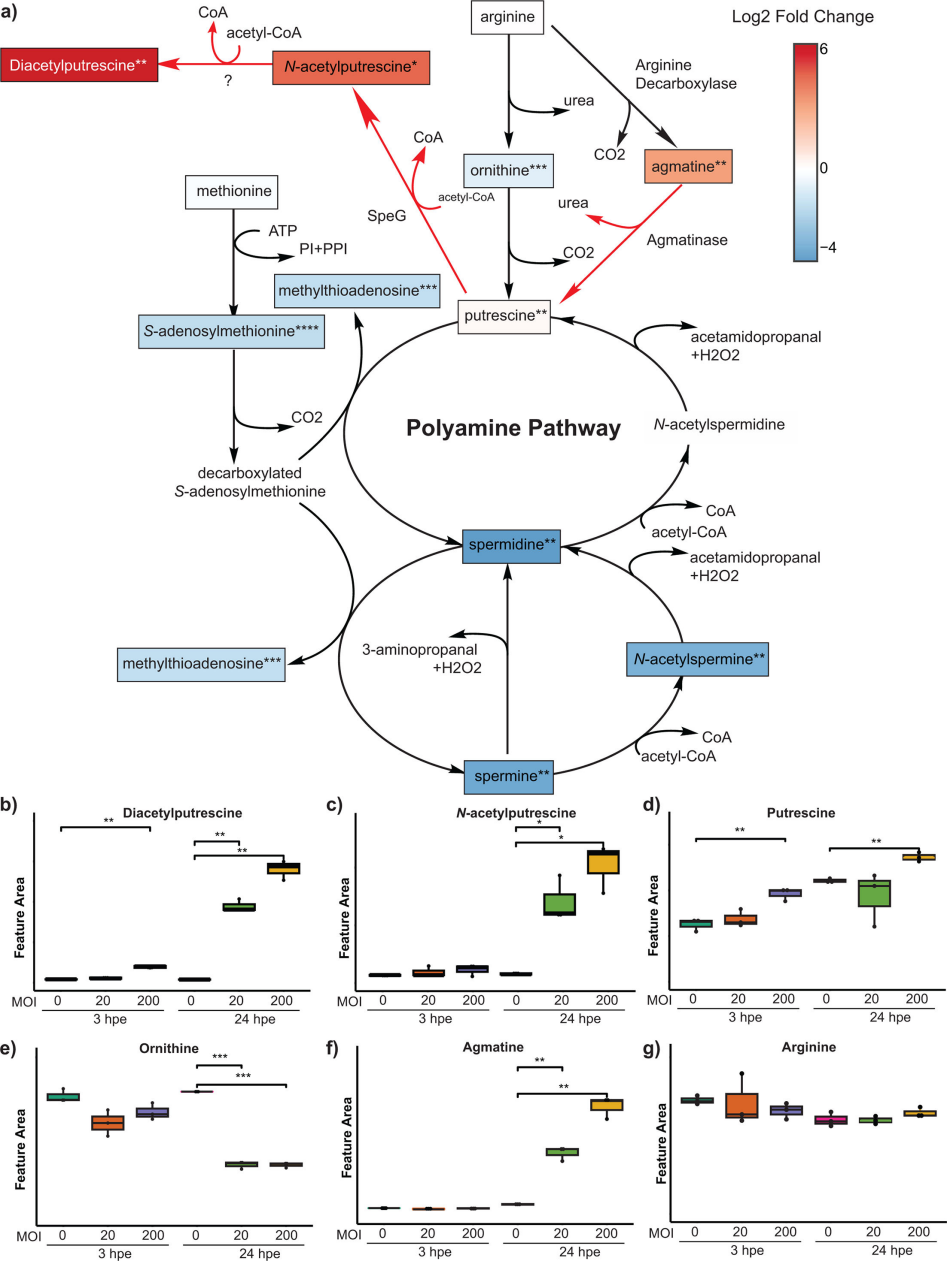
Relative abundances of metabolites in the polyamine metabolic pathway in mock- versus *Bt*-challenged AECs. (**a**) Illustration of the polyamine metabolic pathway. The colors of the boxes indicate the associated log_2_ fold change calculated as (MOI 200, 24 hpe) / (MOI 0, 24 hpe). Metabolites lacking a box were not detected. Bacterial enzymes potentially involved in *N*-acetylputrescine production are included based on KEGG annotations; the reactions that they potentially catalyze are indicated by red arrows. (**b–g**) Boxplots of abundances of polyamine pathway metabolites. Each panel shows the name and the detected abundance of the indicated metabolite. For each culture condition indicated, the distribution of relative abundance data is represented by a boxplot in which the box indicates the central 50% (interquartile range), the vertical lines indicate the range of the remaining data, and the horizontal line indicates the mean value. Asterisks indicate significant differences between groups, as determined by *t*-test and using raw *P*-values. * refers to *P*-value < 0.05, ** refers to *P*-value < 0.01, *** refers to *P*-value < 0.001, and **** refers to *P*-value < 0.0001.

We detected 10 of the 12 key metabolites in the polyamine metabolic pathway and found that in most cases, their levels were altered following *Bt* challenge of AECs (Fig. 3a). Among the detected metabolites, six (ornithine, spermine, spermidine, *N*-acetylspermine, methylthioadenosine, and *S*-adenosylmethionine) were significantly depleted in *Bt*-challenged AECs as compared to mock-challenged AECs (Fig. 3; Fig. S3 and S4). Positively charged polyamines play a vital role in the physiology of both eukaryotes and bacteria. In mammals, these molecules support cell growth, proliferation, transcription, translation, membrane homeostasis, and ion transport, among other cellular processes (37). Depletion of spermine and spermidine has been shown to lead to mitochondria-mediated apoptosis, suggesting their involvement in the inflammatory response in mammalian cells (38). In bacteria, polyamines have been shown to support growth, motility, and biofilm formation; however, an excess of spermidine was found to be detrimental to bacterial growth (39). Thus, in bacteria, the intracellular concentration of these molecules is tightly regulated. This regulation is accomplished primarily through charge neutralization via acetylation and extrusion. In *Bp*, acetylation of polyamines by the bacterial enzyme SpeG was shown to be regulated by sigma factor E during oxidative stress (40). Additionally, in a recent investigation of bloodstream infections by gram-negative pathogens *Pseudomonas aeruginosa* and *Escherichia coli*, inhibition of SpeG was found to enhance bacterial membrane permeability and synergize with vancomycin treatment to reduce proliferation of the bacteria *in vivo* (41). Upon genome analysis of *Bt*, a SpeG homolog was found and has been annotated as SpeG (locus tag: BTH_RS12600) (42). We observed significantly increased levels of both diacetylputrescine and *N*-acetylputrescine during *Bt* challenge of AECs. Thus, polyamine acetylation may be a common mechanism for adaptation to the host environment by these gram-negative pathogens.

Notably, agmatine and putrescine showed the opposite trend of downstream metabolites: they were detected at significantly higher levels in *Bt*-challenged AECs as compared to mock-challenged AECs (Fig. 3d and f). In fact, agmatine was detected exclusively in *Bt*:AEC co-cultures. A number of reports indicate that the pathogen plays an important role in elevating agmatine levels and might also benefit from this increase in agmatine levels (37, 43, 44). For instance, it has been observed that lipopolysaccharide (LPS)-exposed macrophages overproduce agmatine (45). Furthermore, significantly higher levels of agmatine were observed in bronchoalveolar lavage of mouse lungs when exposed to bacteria as compared to LPS, suggesting bacteria contribute to the increase in agmatine. Higher levels of agmatine are also reported during bacterial sepsis; clinical isolates of *Pseudomonas aeruginosa* from cystic fibrosis were previously observed to hyperproduce agmatine, and the presence of agmatine in sputum was found to be correlated with disease severity (46), and this molecule was associated with virulence of several additional human pathogens (47–49). Agmatine-hyperproducing *P. aeruginosa* displayed significantly reduced neutrophil recruitment in these studies, and an increase in agmatine during respiratory infections is positively correlated with activation of inflammation (46). Additionally, hyperproduction of agmatine in *P. aeruginosa* was shown to decrease its susceptibility to cationic antibiotics that target the gram-negative outer membrane as well as hinder IL-8 production by airway epithelial cells in response to bacterial infection (50). Taken together, these observations suggest that agmatine plays a key role in host-pathogen interactions during lung infections and necessitates further investigation into agmatine as a novel target for immune modulation. Thus, our observations support that mammalian cell co-culture metabolomics can highlight relevant metabolic processes that play important physiological roles during infection and enable prioritization for validation and investigations in animal models.

### NAD^+^ metabolism

**Fig 4:**
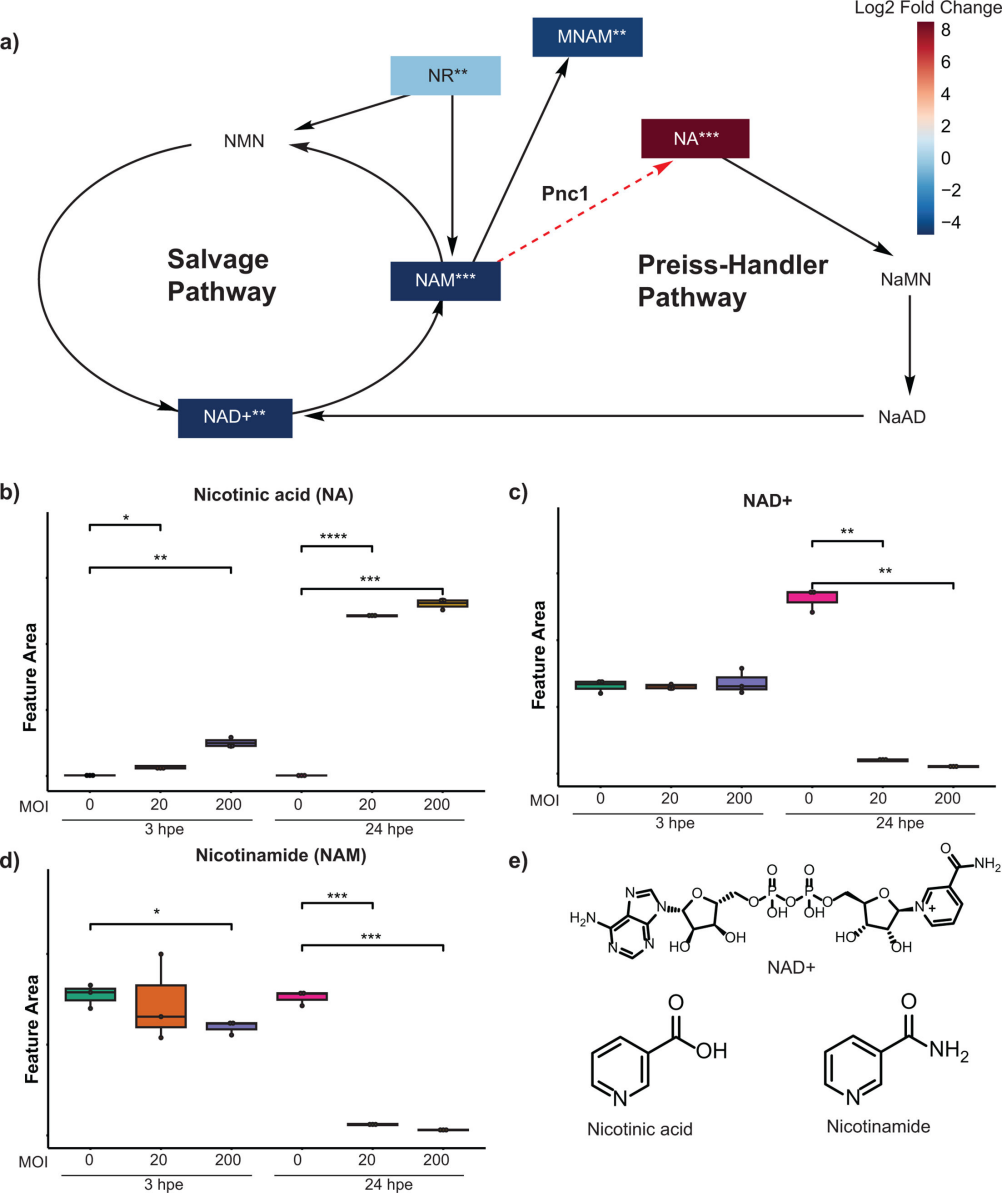
Relative abundances of metabolites in the NAD^+^ salvage and Preiss-Handler pathways in mock- versus *Bt*-challenged AECs. (**a**) Illustration of the NAD^+^ salvage and Preiss-Handler pathways. NR, nicotinamide riboside; NMN, nicotinamide mononucleotide; NaMN, nicotinic acid mononucleotide; NaAD, nicotinic acid adenine dinucleotide; MNAM, methylnicotinamide. The reaction specific to bacteria is indicated by a red dotted line. The colors of the boxes indicate the associated log_2_ fold change calculated as (MOI 200, 24 hpe) / (MOI 0, 24 hpe). Metabolites lacking a box were not detected. (**b–d**) Boxplots of NA, NAD^+^, and NAM abundances. See Fig. 3 legend for format details. * refers to *P*-value < 0.05, ** refers to *P*-value < 0.01, *** refers to *P*-value < 0.001, and **** refers to *P*-value < 0.0001. (**e**) Chemical structures of NA, NAD^+^, and NAM.

We found that *Bt* challenge was associated with significantly reduced levels of NAD^+^ salvage pathway metabolites, as well as significantly increased levels of nicotinic acid (NA) (Fig. 4; Fig. S5), relative to the levels detected in mock-challenge control cultures. Given the importance of NAD^+^ in a wide range of cellular activities (51), these effects are likely to cause broad disruption of host cell functions. One possible explanation for the depletion of NAD^+^ and nicotinamide (NAM) upon *Bt* challenge is based on the fact that in most bacteria, the NAD^+^ pathway involves conversion of NAM to NA, a reaction catalyzed by the nicotinamidase enzyme (PNC1) (52). It is possible that *Bt* disrupts the host cell’s NAD^+^ salvage pathway by promoting conversion of NAM to NA for use in its own metabolism during the intracellular portion of its lifecycle. While mammals do have the capacity to use NA to produce NAD^+^ through the Preiss-Handler pathway, the mouse serum NAM:NA ratio is roughly 10:1, and isotope tracer experiments have shown that the flux of NA to NAD^+^ is minimal in most tissues (53). This suggests that outside of the liver, most mammalian tissues primarily utilize NAM to regenerate NAD^+^. Accordingly, PNC1 could represent a novel therapeutic target for combating *Bt* infection. In fact, a PNC1 inhibitor was shown to reduce proliferation of the human intracellular parasite *Plasmodium falciparum* in red blood cells (33). A similar strategy could prove effective in reducing proliferation of intracellular *Bt* and restoring NAD^+^ metabolism in host cells.

An alternative explanation for our observations is that *Bt* challenge may lead to dysregulation of the host NAD^+^ salvage pathway. There is compelling evidence that the NAD^+^ salvage pathway is often altered in the course of many different bacterial and viral infections, and that this may, in fact, contribute to sepsis (54, 55). For instance, a multi-omics analysis revealed that *Streptococcus pneumoniae* infection was associated with reduced levels of NAD^+^ in the host, as well as altered levels of several proteins involved in the NAD^+^ salvage pathway (56). The authors also found that boosting host NAD^+^ production inhibited bacterial growth in a co-culture model system. Moreover, Li et al. showed that *Bp* infection was associated with dysregulation of three key proteins in the NAD^+^ salvage pathway (NAMPT, NMNAT1, and PNP) in RAW264.7 macrophage cells, indicating that dysregulation of NAD^+^ salvage pathway proteins may be a shared strategy in bacterial pathogenesis (57). Regardless of the underlying mechanism, our results suggest that boosting host NAD^+^ levels might inhibit *Bt* growth during co-culture, a possibility that we intend to investigate in future studies. It will also be important to combine proteomic and metabolomic analyses to identify changes in protein expression that alter the relative abundance of NAD^+^-related metabolites.

### TCA cycle

**Fig 5:**
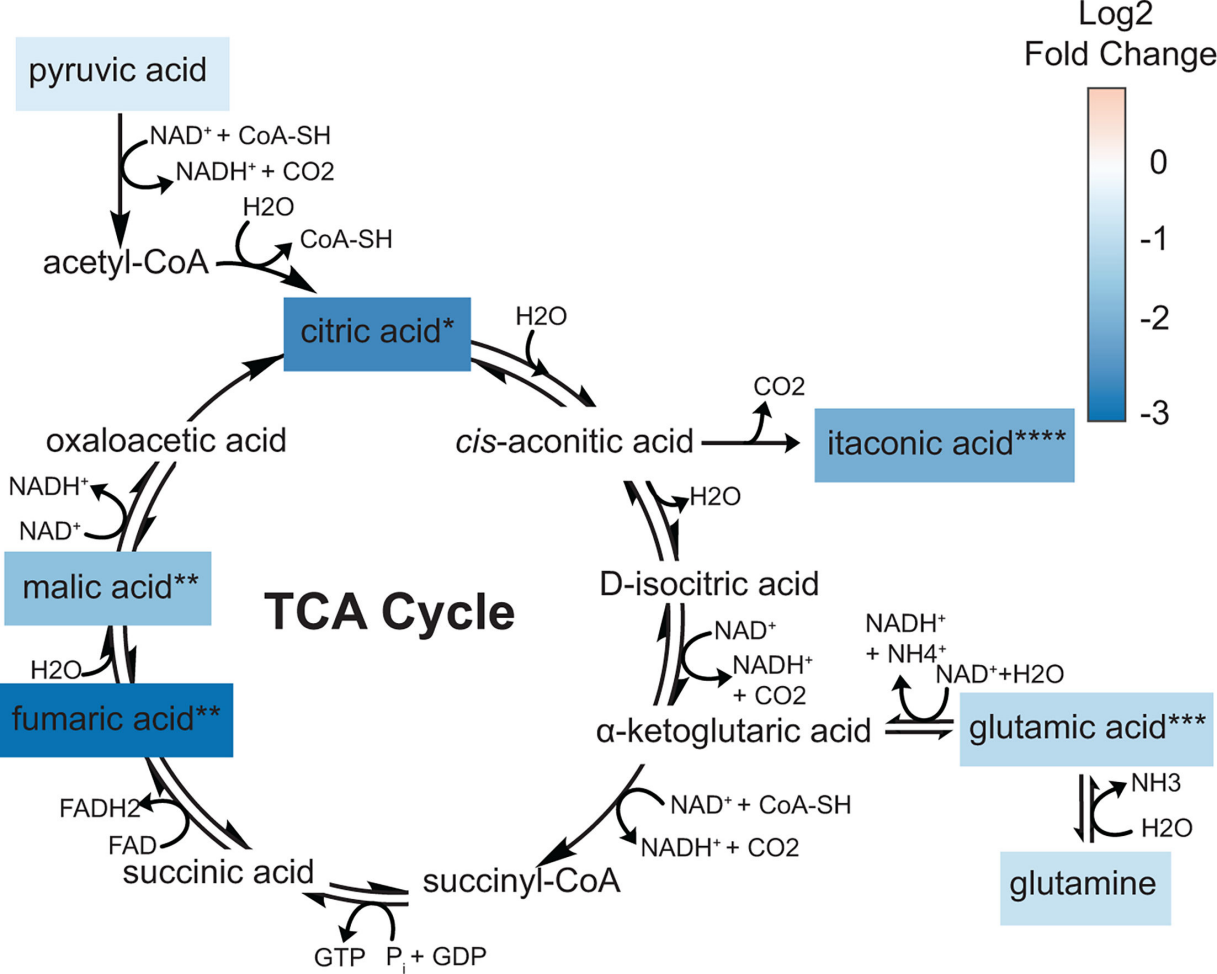
Relative abundances of metabolites in the TCA cycle pathway in mock- versus *Bt*-challenged AECs. The colors of the boxes indicate the associated log_2_ fold change calculated as (MOI 200, 24 hpe) / (MOI 0, 24 hpe). Metabolites lacking a colored box were not detected. Asterisks indicate significant differences between the compared groups, as determined by *t*-test. * refers to *P*-value < 0.05, ** refers to *P*-value < 0.01, *** refers to *P*-value < 0.001, and **** refers to *P*-value < 0.0001.

Disruption of the host’s energy metabolism during *Bt* infection of AECs was further evident in the observed depletion of metabolites involved in the TCA cycle. We found that TCA cycle intermediates (citric acid, fumaric acid, and malic acid) and byproducts (itaconic acid and glutamic acid) were detected at significantly lower levels in *Bt*-challenged AECs as compared to mock-challenged AECs (Fig. 5; Fig. S6). Downregulation of the TCA cycle may indicate a shift of the host’s metabolism to aerobic glycolysis; in oncology, this transition is called the Warburg effect. A “Warburg-like” effect, in which the host shifts from TCA cycle to glycolysis metabolism for energy production, has been widely observed in bacterial infection models, including during the intracellular growth of *Mycobacterium tuberculosis, Legionella pneumophila,* and *Chlamydia trachomatis* (58). Additionally, Li et al. showed through proteomic analyses that glycometabolism pathways were activated in RAW264.7 macrophages during *Bp* infection (57). This infection-associated redirection of the host’s energy-producing metabolism from the TCA cycle to the Warburg-like metabolism may provide the bacterial pathogens with nutritional fatty acids, amino acids, and nucleotides (59, 60). In any case, the mechanisms that mediate this redirection remain poorly understood. Overall, our results provide evidence that *Bt* may derail the host’s mitochondrial function and energy metabolism during infection, a possibility that warrants further investigation.

### Nucleotide bases

**Fig 6:**
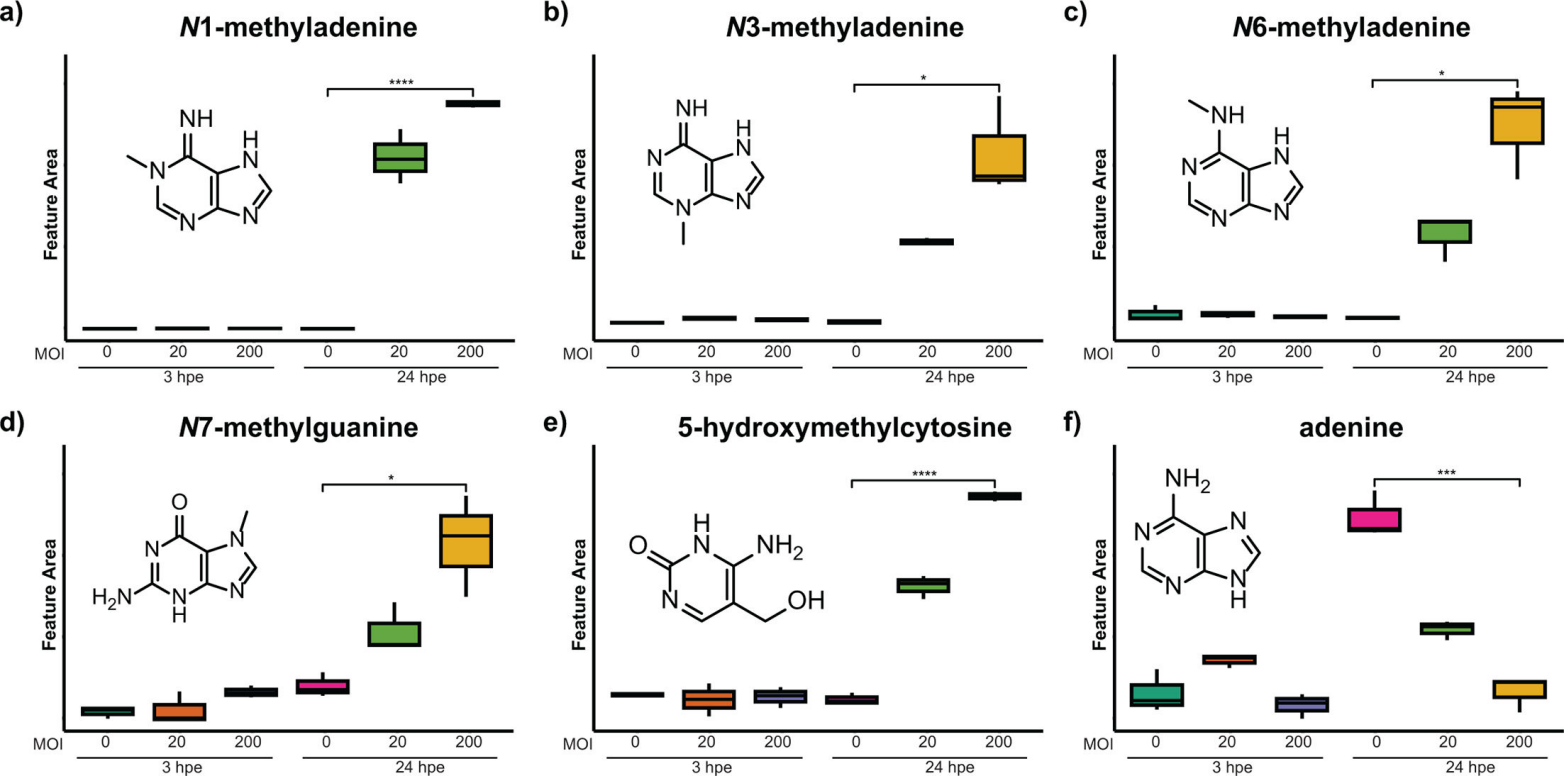
Relative abundances of nucleotide bases (**a–f**) in mock- versus *Bt*-challenged AECs. Each panel shows the name, chemical structure, and detected relative abundance of the indicated nucleotide base. See Fig. 3 legend for format details. * refers to *P-*value < 0.05, ** refers to *P*-value < 0.01, *** refers to *P*-value < 0.001, and **** refers to *P*-value < 0.0001.

A feature at *m/z* 150.078 identified by HCA to be significantly elevated in *Bt*-challenged AECs (as compared to mock-challenged AECs) at 24 hpe (Fig. 6a) was annotated as *N*1-methyladenine, and the annotation was validated with an analytical standard (Fig. S7a). In addition, adenine showed the opposite trend, with consistently lower levels in the *Bt*-challenged AECs at 24 hpe (Fig. 6f). That said, adenine was detected in both mock-challenged AECs (i.e., AEC monocultures) and *Bt*-challenged AECs (i.e., *Bt*:AEC co-cultures), as well as in *Bt* monocultures, as might be expected given its biological function, whereas *N*1-methyladenine was detected only in *Bt*-challenged AECs at 24 hpe, suggesting that methylation of adenine is induced upon *Bt* infection. Through further investigation of features annotated as nucleotide bases, we discovered that a variety of modified nucleotide bases, including *N*3-methyladenine, *N*6-methyladenine, *N*7-methylguanine, and 5-hydroxy-methylcytosine, showed elevated levels in *Bt*-challenged AECs at 24 hpe (Fig. 6b through e). Excretion of *N*3-methyladenine has been noted as a biomarker for DNA damage (61). Thus, a biological source of these metabolites might be the spontaneous depurination of damaged bases or the action of bacterial or mammalian *N*3-methyladenine DNA glycosylases (DNA repair enzymes) (62, 63). This pathway is evolutionarily conserved in bacteria and mammals, such that the detected *N*3-methyladenine could be a manifestation of bacterial and/or mammalian DNA damage during *Bt* infection of AECs. Recently, *N*6-methyladenine was identified as a biomarker of bacterial urinary tract infection and was shown to be produced by the pathogen cultured in filter-sterilized urine (64). Another study identified *N*7-methylguanine to be associated with bloodstream infections (41). Together, these and our metabolomics-based strategies have identified a significant shift in methylated adenine during host-pathogen interactions in different body sites and contexts, including the airway epithelium, the urinary tract, and the blood stream.

### Peptidoglycan biosynthesis and glycometabolism

**Fig 7:**
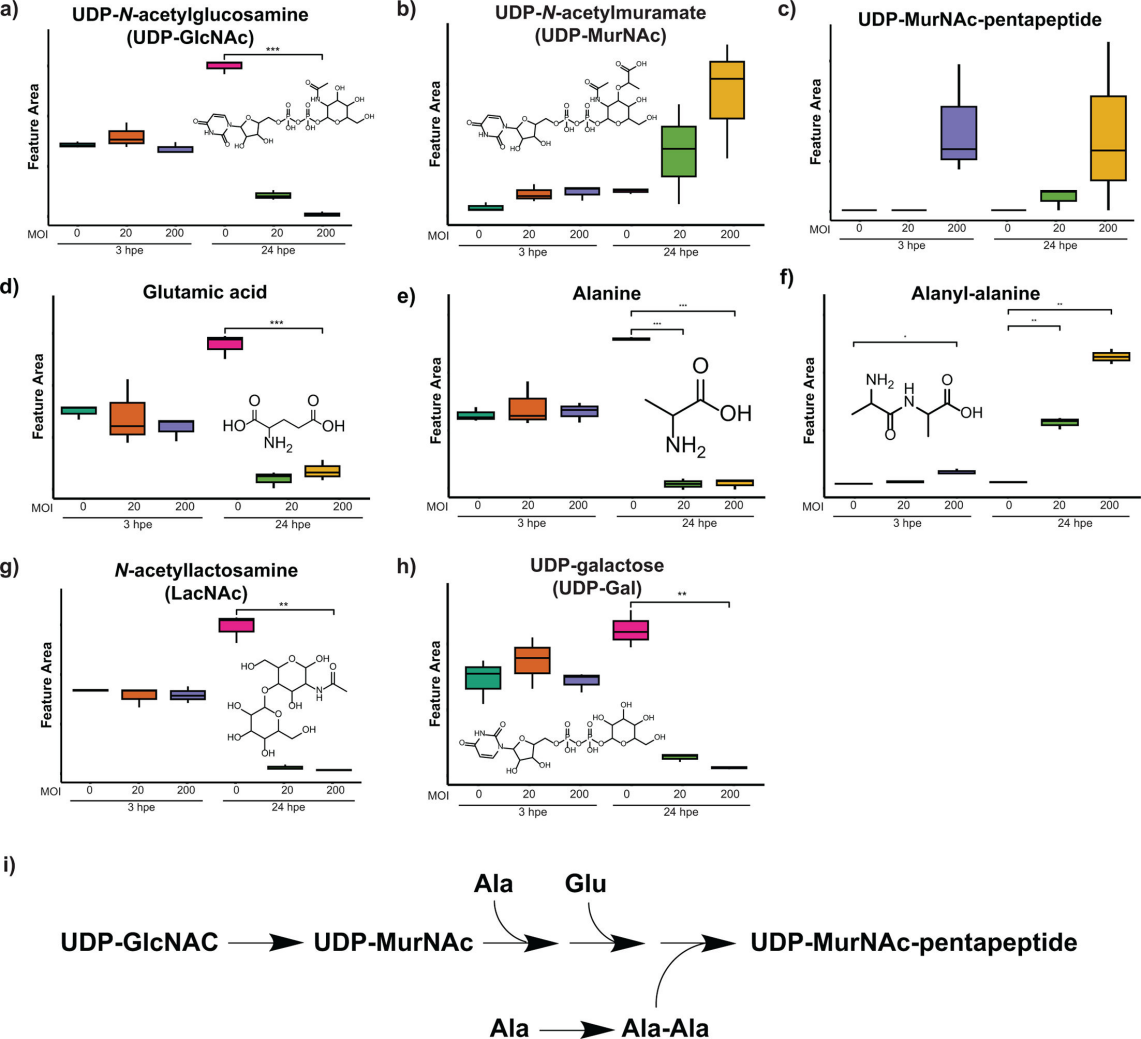
Relative abundances of peptidoglycan intermediates. (**a–h**) Each panel shows the name, chemical structure, and detected relative abundance of the indicated metabolite. See Fig. 3 legend for format details. * refers to *P*-value < 0.05, ** refers to *P*-value < 0.01, *** refers to *P*-value < 0.001, and **** refers to *P*-value < 0.0001. (**i**) Pathway of detected peptidoglycan intermediates.

Peptidoglycan biosynthesis is vital for bacterial survival and is the target of many antibiotics (65). The substrate of the first committed step of peptidoglycan biosynthesis is uridine diphosphate N-acetylglucosamine (UDP-GlcNAc). We detected a significant depletion of this metabolite in *Bt*-challenged AECs (as compared to mock-challenged AECs) at 24 hpe (Fig. 7a). We also detected accumulation of the peptidoglycan intermediates UDP-N-acetylmuramic acid (UDP-MurNAc) (Fig. 7b) and UDP-MurNAc-pentapeptide (Fig. 7c) only in *Bt*-challenged AECs at 24 hpe, with both annotations supported by shared MS^2^ peaks with UDP-GlcNAc (Fig. S9). Additionally, peptidoglycan biosynthesis utilizes glutamic acid and alanine as precursors, and we found that both were depleted in *Bt*-challenged AECs at 24 hpe (Fig. 7d and e); and that an intermediate, alanyl-alanine, accumulated only in *Bt*-challenged AECs (Fig. 7f). These results suggest that *Bt* utilizes host metabolites such as UDP-GlcNAc, glutamic acid, and alanine for peptidoglycan biosynthesis, causing their depletion, as well as concomitant accumulation of peptidoglycan intermediates (UDP-MurNAc, UDP-MurNAc-pentapeptide, and alanyl-alanine), in *Bt*:AEC co-cultures.

UDP-GlcNAc is also an important substrate and precursor for glycosylation of proteins and lipids, which is one of the most common post-translational modifications and is crucial for many cellular functions, including protein maturation, stabilization, and subcellular localization, signal transduction, and proliferation (66, 67). In fact, glycosylation has been implicated as a key signal in innate immune responses, and altered flux in glycosylation has been observed during immune activation and apoptosis (68, 69). A proteomics study of *Bp* infection of macrophages revealed abnormal glucose metabolism (Warburg-like metabolism) and reduced O-GlcNAcylation levels (57), consistent with the observations made in our analysis of *Bt* infection of AECs via untargeted metabolomics; this parallel provides further indication that *Bp* and *Bt* share pathogenesis features and serves as another example of the value in using multiple omics analysis platforms to identify disease-relevant processes and pathways.

In addition to UDP-GlcNAc, we observed depletion of *N*-acetyl-lactosamine (LacNAc) and UDP-galactose (UDP-Gal) in *Bt*-challenged AECs at 24 hpe (Fig. 7g and h). LacNAc is an important component of many glycoprotein carbohydrate chains (70), and UDP-Gal is an intermediate in the biosynthesis of polysaccharides. Depletion of UDP-GlcNAc, LacNAc, and UDP-Gal, together with the shift from the TCA cycle to Warburg-like metabolism, is a clear indication that *Bt* infection causes a broad disruption of glycometabolism in the host, consistent with observations made in proteomics studies of *Bp* infection (57).

### Tryptophan metabolism

**Fig 8:**
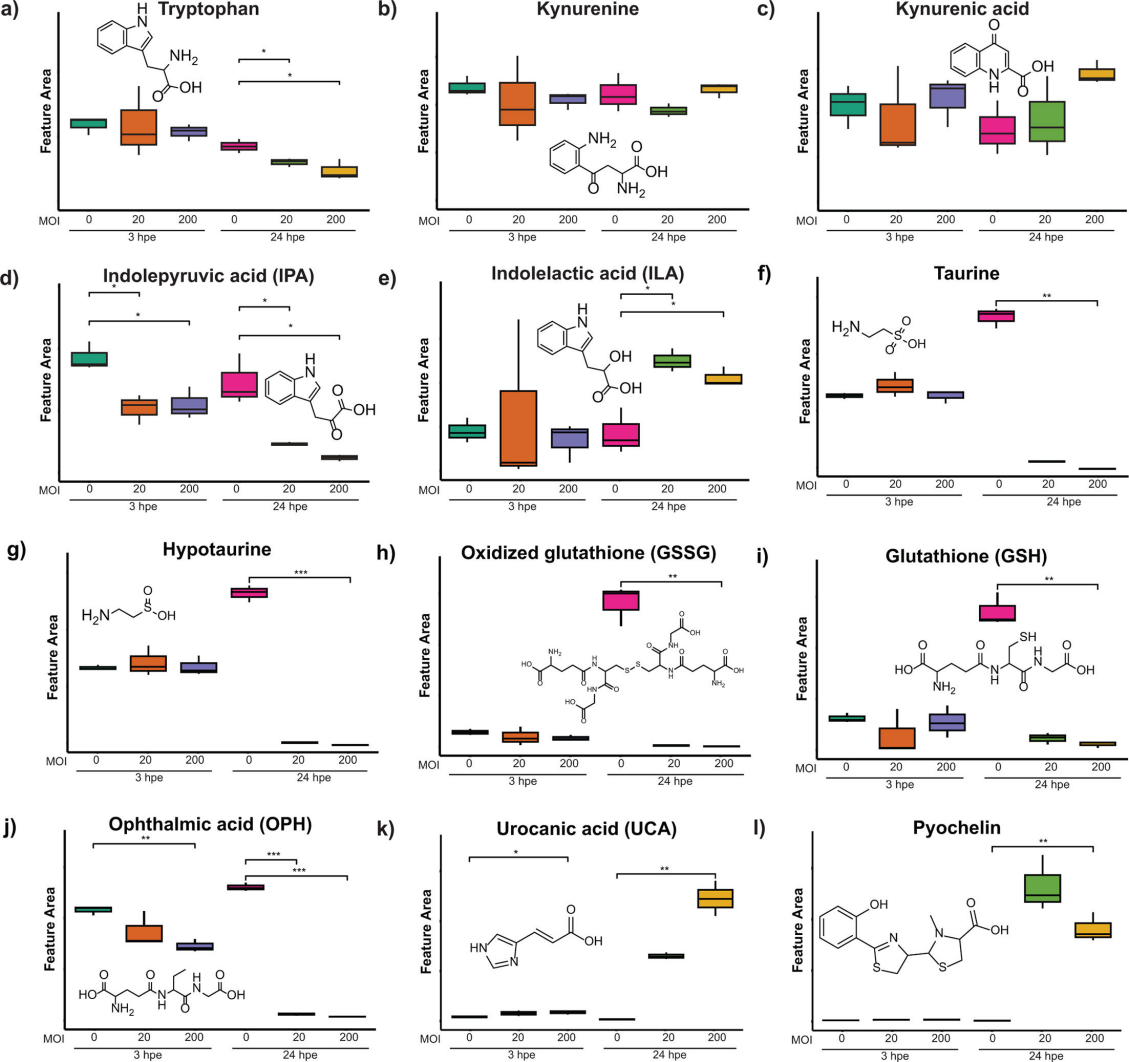
Relative abundances of metabolites in mock- versus *Bt*-challenged AECs. Each panel (**a–l**) shows the name, chemical structure, and detected relative abundance of the indicated metabolite. See Fig. 3 legend for format details. * refers to *P*-value < 0.05, ** refers to *P*-value < 0.01, *** refers to *P*-value < 0.001, and **** refers to *P*-value < 0.0001.

We observed a significant depletion of tryptophan in *Bt*-challenged (as compared to mock-challenged) AECs at 24 hpe (Fig. 8a). The intricacy of host-microbe interactions around tryptophan metabolism has been studied primarily in the gut. In mammals, tryptophan is an essential amino acid that has three metabolic fates: incorporation into proteins, breakdown into kynurenine, and conversion into indole and its derivatives (71). The microbes can also metabolize tryptophan in the gut. The interplay between host- and microbe-driven tryptophan metabolism has been implicated in immune tolerance and maintenance of gut microbiota (72). We found that metabolites produced by the kynurenine pathway (kynurenine and kynurenic acid), which metabolizes most free tryptophan and plays a key role in NAD^+^ biosynthesis, did not show significant changes in abundance as a function of *Bt* infection (Fig. 8b and c). On the other hand, indole derivatives showed significant changes in abundance in *Bt*-challenged AECs, with indolepyruvic acid detected at reduced levels (Fig. 8d) and indolelactic acid detected at elevated levels (Fig. 8e). These results suggest that *Bt* alters host indole metabolism during infection.

### Taurine and related metabolites

Taurine, a sulfur-containing cysteine metabolite, was found to be significantly lower in *Bt*-challenged AECs (as compared to mock-challenged AECs) at 24 hpe (Fig. 8f). The related metabolite, hypotaurine, showed a similar decrease in abundance in *Bt*-challenged AECs at 24 hpe (Fig. 8g). Taurine contributes to mammalian immune responses by inducing the release of IL-35, inhibiting oxidative stress, promoting release of pro-inflammatory cytokines, and stimulating lymphocytes, neutrophils, and macrophages to defend against pathogens (73–76). The pathogen used in our study, *B. thailandensis* strain E264 (*Bt*), is capable of utilizing taurine as a source of sulfur, and pathogenic *Bp* strains have been found to exhibit a significantly enhanced ability to utilize taurine efficiently compared to *Bt* (77). Furthermore, in a study investigating the interactions of *Burkholderia cenocepacia* with bronchial cells, the metabolism of sulfur, taurine, and hypotaurine was found to be upregulated (78). These observations suggest that *Bt* directly depletes taurine and hypotaurine levels in AECs during infection and, in doing so, deprives the AECs of their immunological benefits.

### Glutathione (GSH) and related metabolites

We detected significant depletion of GSH, oxidized glutathione (GSSG), and the related metabolite ophthalmic acid (OPH) in *Bt*-challenged AECs as compared to mock-challenged AECs (Fig. 8h through j). GSH plays an important role in a variety of mammalian cell functions, including disulfide bond formation, iron-sulfur cluster formation, metabolite transport, redox balance, and cellular signaling (79), so its depletion during *Bt* infection of AECs could result in broad dysregulation of host metabolism and responses to oxidative and nitrosative stress. Depletion of GSH, GSSG, and OPH during *Bt* infection of AECs could result from depletion of glutamic acid (Fig. 7d), as it is a precursor required for biosynthesis of all three of these metabolites.

### Accumulation of urocanic acid (UCA)

We detected significant accumulation of UCA in *Bt*-challenged AECs as compared to mock-challenged AECs, at both 3 hpe and 24 hpe (Fig. 8k). UCA biosynthesis is accomplished through deamination of histidine by histidine ammonia-lysase (histidase), which constitutes the initial step of the histidine degradation pathway (80). UCA is then hydrolyzed by urocanate hydratase (urocanase) to generate imidazolonepropionate, which is further processed in subsequent steps to ultimately produce glutamic acid. Orthologs of the genes encoding histidase (*hutH*) and urocanase (*hutU*) are present in the *B. thailandensis* E264 (*Bt*) genome, and mammalian cells can produce UCA as well, so the source of UCA in *Bt*:AEC co-cultures remains to be determined. There is mounting evidence that UCA produced by the host (and/or histidine produced by the host and then converted to UCA by the bacteria) can influence expression of *hut* genes, as well as genes encoding virulence factors, in gram-negative pathogens (80). In addition, UCA has been shown to have anti-inflammatory and immunosuppressive properties (81). Thus, UCA may directly mediate chemical interaction between *Bt* and AECs, and its accumulation over the course of infection likely holds important implications for both pathogen and host.

### Pathogen-specific metabolites

Several ornithine lipids (OLs) and a lyso-OL were detected at elevated levels in *Bt*-challenged AECs as compared to mock-challenged AECs (Fig. S10). OLs are phosphorus-free bacterial membrane lipids that confer stress resistance (82). Lyso-OLs are precursors for biosynthesis of OLs; they consist of a 3-hydroxy fatty acid linked to the alpha amino group of an ornithine amino acid through an amide bond. OLs have been shown to affect infection dynamics by stimulating the immune system (83) and increasing bacterial persistence and antibiotic resistance (84). OLs can be hydroxylated or non-hydroxylated, and disruption of genes required for hydroxylation of OLs has been shown to reduce stress resistance (85). Our results indicate that as *Bt* infection of AECs progresses from 3 hpe to 24 hpe, there is a shift in relative abundance from hydroxylated to non-hydroxylated OLs (Fig. S10e). This observation highlights the need for further study of the role of OLs in *Burkholderia* infection, particularly as a function of hydroxylation state.

Additionally, several specialized bacterial metabolites previously shown to be produced by *Bt* were detected during infection of AECs. These included several HMAQs, an HMAQ-*N-*oxide, an acylhomoserine lactone, pyochelin, hopanoids, acybolins, and bactobolins (Table S1; Fig. 8l). Several of these secondary metabolites have been demonstrated to exhibit antimicrobial activity against gram-positive and/or gram-negative bacteria (86–88). While HMAQs and their *N-*oxide counterparts are known modulators of quorum sensing, a bacterial cell signaling system that controls expression of many virulence factors, their exact role in pathogenesis is largely uncharacterized (89). Bactobolin A and acybolin A are structurally related compounds that are produced by the same biosynthetic gene cluster and have been implicated in mediating host-pathogen interactions in other infection models (90, 91). Pyochelin is a siderophore that mediates iron acquisition in the iron-limited environment of the host, and thus constitutes an important virulence factor (82). Hopanoids such as bacteriohopanetetrol cyclitol ether have been shown to enhance bacterial stress resistance (92). Detection of these known and suspected bacterial virulence factors intracellularly in our model system suggests that they may be involved in the intracellular stage of *Bt* infection.

### Conclusions

Metabolic profiling of the intracellular bacterial pathogen *Bt* during infection of AECs revealed the complexity of its interactions with the host in even a relatively simple model system. Our results indicate that *Bt* can radically disrupt fundamental host functions such as energy metabolism and amino acid utilization, as well as several pathways implicated in host defense and/or immunometabolic cross-talk between pathogen and host (e.g., the polyamine pathway, NAD^+^ salvage pathway, and TCA cycle). The changes in central metabolism during *Bt* infection of AECs appear to indicate the pathogen’s ability to simultaneously utilize host nutrients and disrupt important host metabolic processes. We anticipate that further investigation of these changes and their outcomes will reveal new opportunities for therapeutic intervention against intracellular bacterial pathogens such as *Bp*. As an example, inhibition of bacterial nicotinamidase (PNC1) or polyamine acetyltransferase (SpeG) is predicted to limit intracellular proliferation and thus would seem promising targets for therapeutics development (Fig. 8).

Our results also highlight the need for detailed analysis of the molecular mechanisms by which metabolic signaling is regulated during infection and make a strong case for the *Bt*:AEC co-culture model system as a useful test bed for such studies. Development of comparable models that use other host cell types of relevance to *Burkholderia* infection (e.g., macrophages, dendritic cells, and neutrophils) should enable comparative analyses that reveal cell type-specific versus agnostic interactions, pathogenesis strategies, and intervention opportunities. Moreover, the development of infection models that recapitulate the complex multicellular organization of tissues (e.g., comprised of multiple cell types and cultivated at an air-liquid interface), lung-on-a-chip as well as organoid models should enable study of infection-associated changes to shared metabolic pools, proximal versus distal effects of metabolite production/depletion, and many other potentially critical determinants of pathogen and host fate in the context of infection.

## MATERIALS AND METHODS

### Culture of *Burkholderia* bacteria and airway epithelial cells

*B. thailandensis* E264 (*Bt*) (a kind gift from Colin Manoil [University of Washington, Seattle, WA, USA]) and murine AEC line LA-4 (American Type Culture Collection [ATCC, Manassas, VA, USA]) were cultured individually and together as previously described (15). Briefly, *Bt* bacteria were propagated in monoculture in LB (Teknova, Hollister, CA, USA) at 37°C, while AECs were propagated in monoculture in F12K medium (ATCC) supplemented with fetal bovine serum (FBS; ATCC) (15% vol/vol) and penicillin/streptomycin antibiotics (Thermo Fisher Scientific, Waltham, MA, USA) (1% vol/vol) at 37°C in an atmosphere of 5% CO_2_. For co-culture (infection) experiments, the AECs were recovered into F12K-based culture medium lacking antibiotics and further propagated at 37°C in 5% CO_2_ for 24 h, at which point *Bt* was recovered into the same culture medium (via centrifugation of the LB culture followed by resuspension of the pelleted bacteria in F12K-based culture medium lacking antibiotics) and added to the AECs to achieve an MOI of either 20 or 200. Actual MOIs were verified to be within ±20% of the intended MOIs, through enumeration of colony-forming units (CFU) in the *Bt* dosing material used to generate co-cultures. Control conditions included AEC monocultures (mock-challenged AEC cultures), in which *Bt* was not added to the AECs (referred to as the MOI 0 condition); and *Bt* monocultures (*Bt*-only cultures), in which *Bt* (already resuspended in F12K-based culture medium) was added to Petri dishes containing only F12K-based culture medium (i.e., lacking AECs). Each co-culture and control culture was replicated in six Petri dishes, all of which were incubated at 37°C in 5% CO_2_ for 1.25 h to allow for opportunity for internalization of *Bt* by the AECs in the co-cultures. After this internalization period, the conditioned culture medium (CCM) (including any *Bt* not attached to or internalized by the AECs in co-cultures) was removed through aspiration, the dish-adhered AECs were washed once with phosphate-buffered saline (PBS) to remove residual free *Bt*, and the PBS was replaced with F12K-based culture medium supplemented with three antibiotics (kanamycin, gentamicin, and imipenem) that are excluded from mammalian cells, to prevent proliferation or additional internalization of any remaining extracellular *Bt* without inhibiting the growth of already internalized (intracellular) *Bt*. Mock-challenged AEC cultures were treated in the same way, whereas for *Bt*-only cultures, the CCM was left in place and not supplemented with antibiotics. At this point (defined as 0 h post-exposure [0 hpe]), the co-cultures and control cultures were returned to incubation at 37°C in 5% CO_2_, with one set of three biological replicates harvested at 3 hpe (i.e., 3 h later) and the other at 24 hpe. This experiment was carried out three times in total. Additional studies were carried out to verify that the *Bt*:AEC co-culture protocol led to internalization of *Bt* by the AECs, as evident from detection of intracellular CFU. In these experiments, *Bt*:AEC co-cultures were established as described above, and at predefined time points (3, 6, 12, 18, 24, 36, and 48 hpe), the CCM was removed; the dish-adhered AECs were washed once with PBS and then selectively lysed through addition of 0.5% saponin in PBS; the lysate was recovered using a cell scraper and P-1000 pipetman; serial 1:10 dilutions of the lysate in PBS were plated on LB agar; and the plates were incubated at 37°C for 48 h for enumeration of viable *Bt* bacteria (CFU). The results confirmed that *Bt*:AEC co-culture at an MOI of either 20 or 200 led to internalization of *Bt* by 3 hpe (Fig. S1). A transient decrease in viable counts by 6 hpe was followed by a modest increase by 24 hpe, indicating that *Bt* was capable of multiplying within the AECs during infection. We observed a decrease in viable counts at later time points (36 and 48 hpe), which might be due to cytotoxic effects that cause release of intracellular *Bt* to the CCM. However, we verified that cytotoxicity levels were ≤25% in all experimental conditions used for metabolomic profiling studies (i.e., MOI 20 or 200 in combination with 3 or 24 hpe), as determined in pilot experiments using standard techniques (LDH release and trypan blue exclusion assays).

### Extraction methods

At the time of harvest, the CCM was removed from each culture and replaced with pre-chilled 80% methanol (vol/vol in water). Two serial extractions were carried out, and each extract was dried *in vacuo* and stored at −80°C, as previously described (15). The first extract from each culture was resuspended in 65 µL of 80% methanol containing stable isotope-labeled internal standards (hypoxanthine-^13^C_5_ [39.7 µM], L-arginine HCl-^13^C_6_ [808 µM], hippuric acid-(benzoyl-d_5_) [256 µM], and L-methionine-(methyl-^13^C,d_3_) [212 µM]; Cambridge Isotope Laboratories) by vortexing briefly and sonicating for 5 min at 4°C, and then centrifuged at 16,160 × *g* for 1 min. To prevent dilution, the supernatant derived from the culture’s first extract was then used to resuspend the culture’s second extract using the same process, and the final supernatant (referred to as the culture’s summed extract, as it contained all metabolites recovered from both of its serial extracts) was stored at −80°C.

### Mass spectrometry data acquisition

Chromatographic separation of the components within each culture’s summed extract was achieved using a Waters Acquity UHPLC system (Waters, Milford, MA) comprised of a Waters Acquity UHPLC BEH Amide column (150 × 2.1 mm, 1.7 µm) interfaced with an ultra-high-resolution Thermo Fisher Scientific Q Exactive HF mass spectrometer equipped with a Heated Electrospray Ionization source in both positive and negative modes. The column temperature was set at 40°C, and the sample injection volume was 1 µL. Elution was performed using mobile phase A (80:20 H_2_O/MeCN containing 0.1% formic acid and 10 mM ammonium formate, pH 3.3) and phase B (acetonitrile, containing 0.1% formic acid) at a flow rate of 0.4 mL/min. The gradient started at 5% A and 95% B for 0.5 min, followed by a linear increase of solvent A to 60% over 7.5 min, held at 60% A for 1.4 min, decreased linearly to 5% A for 0.1 min, and held for another 2.5 min. Mass spectra full scans were performed in both positive and negative ion modes in ranges *m/z* 80 to 1,200 Da with a resolution of 30,000, a maximum injection time (MIT) of 100 ms, and automatic gain control (AGC) of 1 × 10^6^. For MS^2^ data acquisition in both positive and negative ion modes, the five most intense ions per MS^1^ spectrum were selected for fragmentation over a mass range of *m/z* 80 to 1,200 at a resolution of 15,000, MIT of 50 ms, and AGC of 2 × 10^5^. Stepped normalized collision energy was set at 25, 35, and 45. For each scan, the AGC was set at 2 × 10^5^, and the MIT was 50 ms. The dynamic exclusion of precursor ion masses to suppress repeated peak fragmentation was set at 3.0 s.

### Mass spectrometry data processing and statistical analysis

For data analysis and feature annotation, Compound Discoverer software (v.3.3) (93) was used with a workflow template, “Untargeted Metabolomics using Online Databases, mzLogic, and Molecular Networks.” The LC/MS-acquired raw data were uploaded, filtered, and the retention times aligned. Then, detection and grouping of unknown compounds were carried out, followed by compound prediction and annotation, with tentative compound identification performed by comparing experimental data with information available in databases including ChemSpider, mzCloud, Mass Lists, and mzVault. Further support for proposed annotations was provided by relevant literature searches, through manual inspection, and by comparison with published MS^2^ spectra. We detected 5,123 features in positive mode and 2,305 features in negative mode, with 235 features detected in both modes based on shared molecular weight and retention time.

The pre-processed data were downloaded from Compound Discoverer for use in statistical analyses, including PCA and HCA. These analyses were performed using MetaboAnalyst 5.0 and 6.0, removing duplicate features and redundant adducts before applying log transformation (94). The raw data files can be found at https://massive.ucsd.edu with the identifier MSV000098746.

## SUPPLEMENTAL MATERIAL

Supplemental material msystems.00611-25-s0001.docx Fig. S1-S10 and Table S1.

ASM does not own the copyrights to Supplemental Material that may be linked to, or accessed through, an article. The authors have granted ASM a non-exclusive, world-wide license to publish the Supplemental Material files. Please contact the corresponding author directly for reuse.
