# Supplementary material for: Metabolomic profiling of Burkholderia thailandensis infection of airway epithelial cells provides insights into potential therapeutic targets
Source: mSystems. 2025 Oct 31;10(11):e00611-25. doi: 10.1128/msystems.00611-25 (PMC12625709; doi:10.1128/msystems.00611-25)
Supplement: Supplemental material — Fig. S1-S10 and Table S1. [file msystems.00611-25-s0001.docx]

**Title:** Metabolomic profiling of *Burkholderia thailandensis* infection of airway epithelial cells provides insights into potential therapeutic targets

**Short title:** Changes in central metabolism upon *Burkholderia* mammalian cell co-culture

Daniel J. Hicks^a#^, Nicole Aiosa^a#^, Anupama Sinha^b^, Olakunle A. Jaiyesimi^a^, Steven S. Branda^b^*, Neha Garg^a,c^*

^a^School of Chemistry and Biochemistry, Georgia Institute of Technology, 950 Atlantic Drive, Atlanta, Georgia 30332-2000, United States

^b^Biotechnology & Bioengineering, Sandia National Laboratories, 7011 East Avenue, Livermore, CA-94550, USA

^c^Center for Microbial Dynamics and Infection, Georgia Institute of Technology, 311 Ferst Drive, ES&T, Atlanta, GA 30332, USA

^#^Equal contribution first author

*To whom correspondence should be addressed, email address: neha.garg@chemistry.gatech.edu

Keywords: infection culture metabolomics, melioidosis, *Burkholderia*, airway epithelial cells, HILIC

**Table of Contents**

Fig. S1.  Enumeration of viable intracellular bacteria in Bt:AEC co-cultures……………………… **3**

Fig. S2. Heatmap of the top 50 features for hierarchical clustering analysis (HCA) of negative mode data.**4**

Fig. S3: Boxplots of relative abundances of polyamine pathway metabolites in mock- *versus* Bt-challenged AECs ……………………………………….……………………………………….………**5**

Fig. S4. MS^2^ mirror plots of polyamine pathway metabolites…………………………………....**6−7**

Fig. S5. MS^2^ mirror plots of NAD+ salvage pathway metabolites………………………..…….……**8**

Fig. S6. MS^2^ mirror plots and boxplots of TCA cycle metabolites………………………………**9−11**

Fig. S7. MS^2^ mirror plots of methylated nitrogenous base metabolites………………...…………**11**

Fig. S8. MS^2^ mirror plots of additional metabolites…………………………………….………..**12−13**

Fig. S9. Annotation of peptidoglycan intermediates................……………….……………………**13**

Fig. S10. Statistical and spectral analyses of ornithine lipids................…………….…….........**14**

Table S1. Metabolite detection and annotation………………………………………….………**15−16**


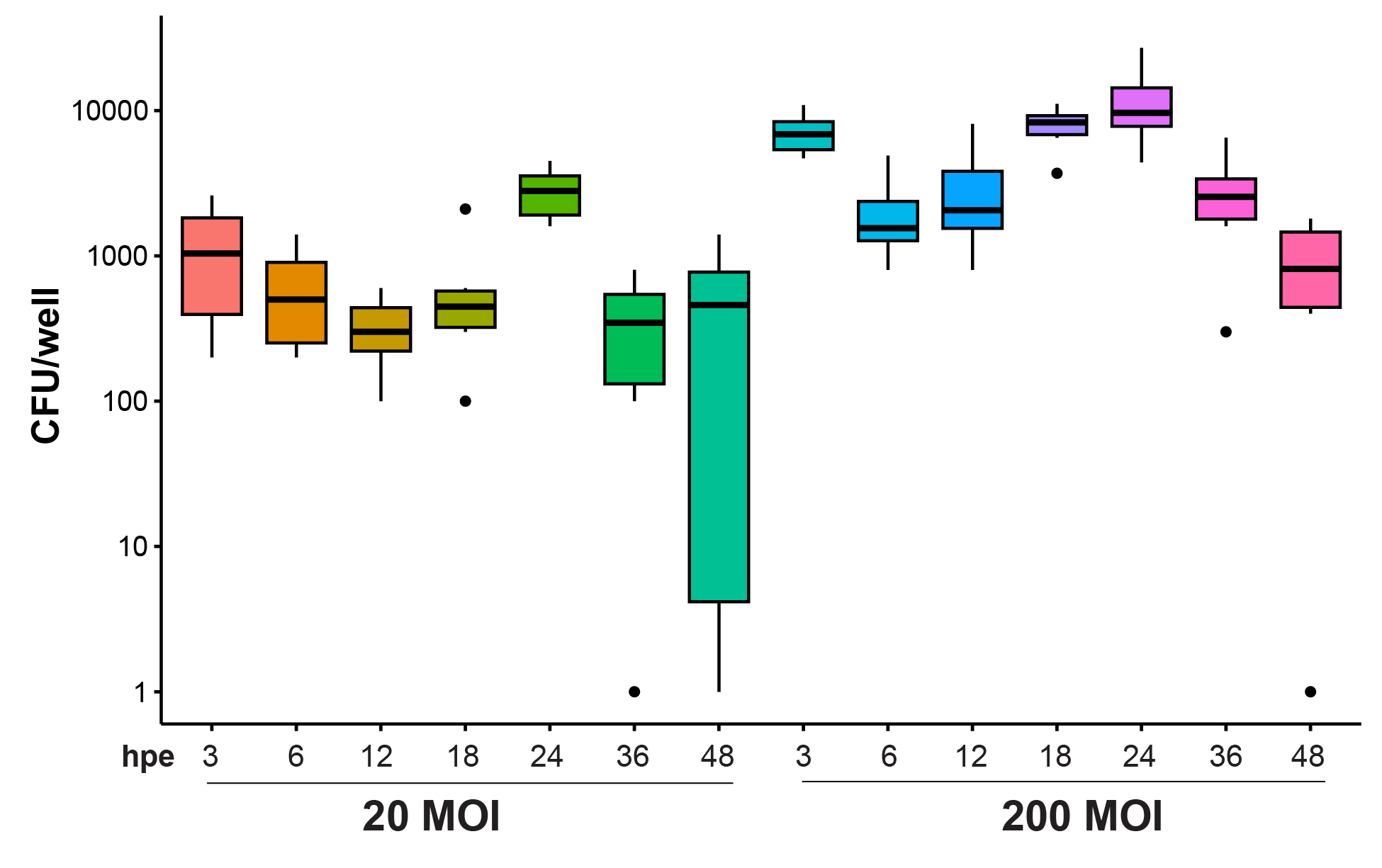


**Supplementary Figure 1.  Enumeration of viable intracellular bacteria in Bt:AEC co-cultures.** Bt bacteria were added to AECs to achieve an MOI of either 20 or 200. After an internalization period of 1.25 h, the adherent AECs were washed once with PBS and then cultured in F12K-based medium supplemented with antibiotics (to prevent growth of any residual extracellular bacteria). At the pre-defined timepoints indicated the AECs were washed once with PBS and then selectively lysed through addition of 0.5% saponin in PBS. The lysates were recovered, and serial 1:10 dilutions in PBS were plated on LB agar for CFU analysis. Box plots of CFU counts from 6 replicate Bt:AEC co-cultures are shown.


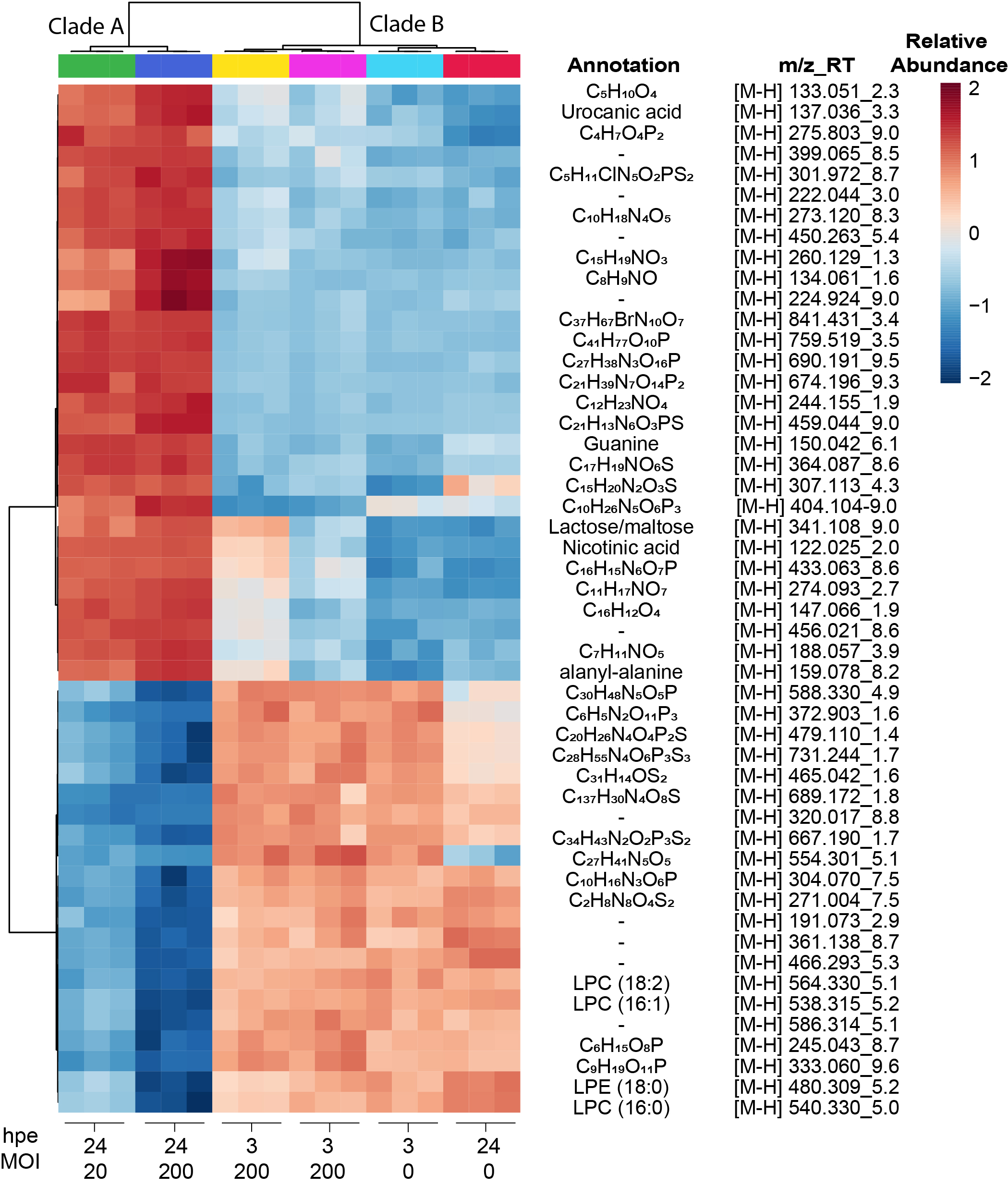


**Supplementary Figure 2**. **Heatmap of the top 50 features for hierarchical clustering analysis (HCA) of negative mode data**. The relative abundance of each feature, along with its annotation or proposed chemical formula (where possible), is shown.


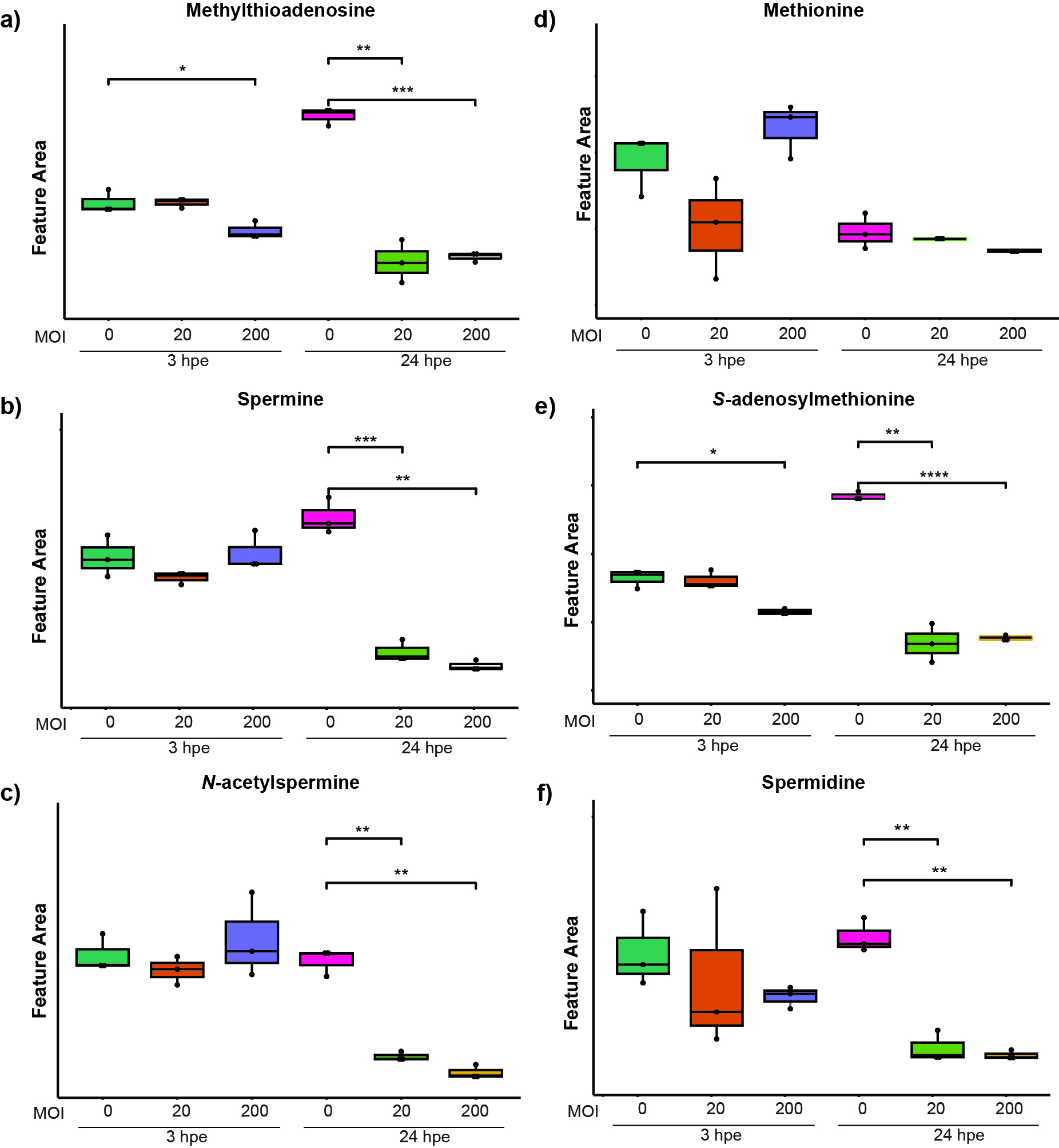


**Supplementary Figure 3. Boxplots of relative abundances of polyamine pathway metabolites in mock- *versus* Bt-challenged AECs*.*** Each panel shows the name and detected abundance of the indicated metabolite. For each culture condition indicated, the distribution of relative abundance data is represented by a boxplot in which the box indicates the central 50% (interquartile range), the vertical lines indicate the range of the remaining data, and the horizontal line indicates the mean value. Asterisks indicate significant differences between the compared groups, as determined by *t* test. * refers to *p*-value < 0.05, ** refers to *p*-value < 0.01, *** refers to *p*-value < 0.001, **** refers to *p*-value < 0.0001.


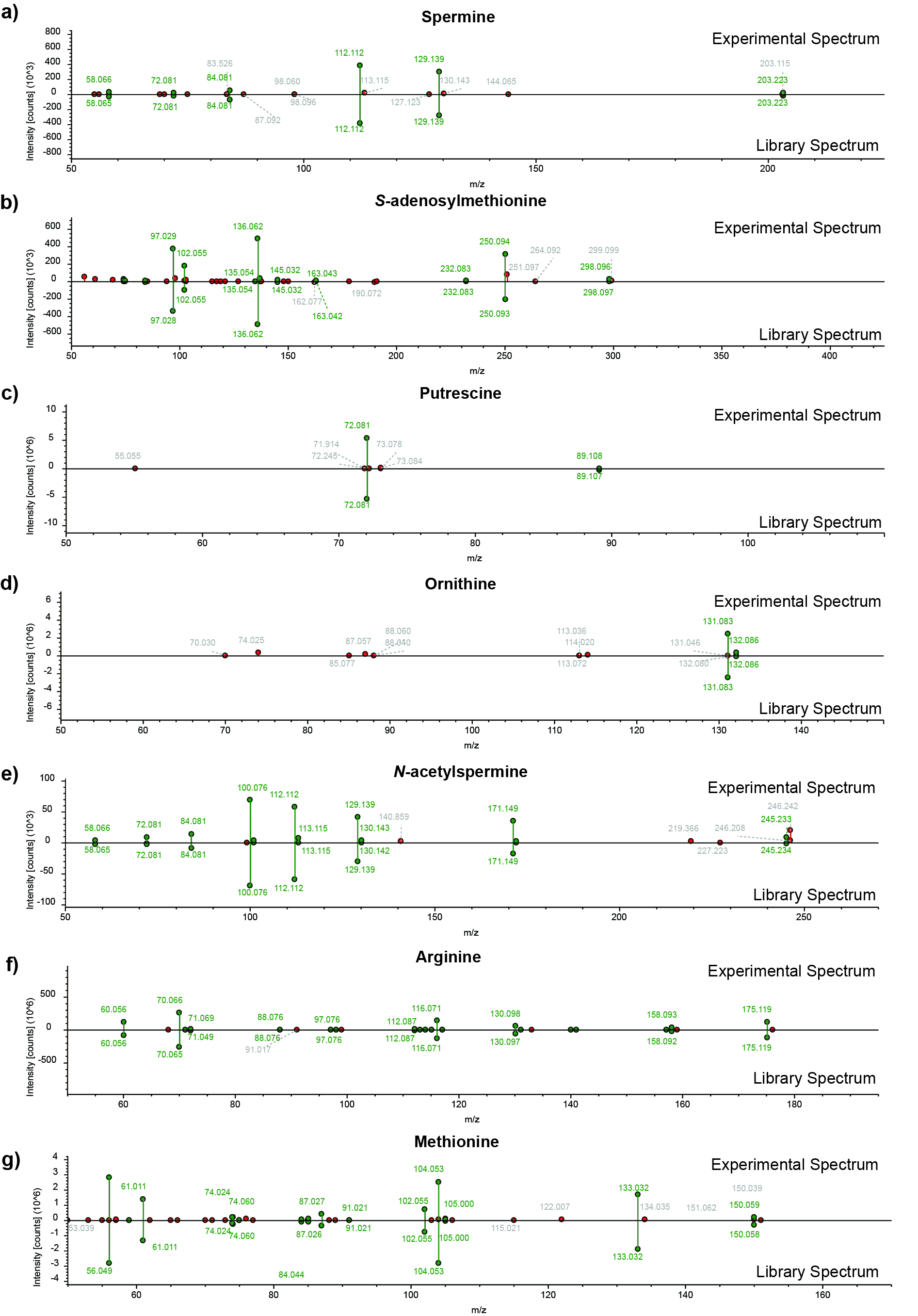

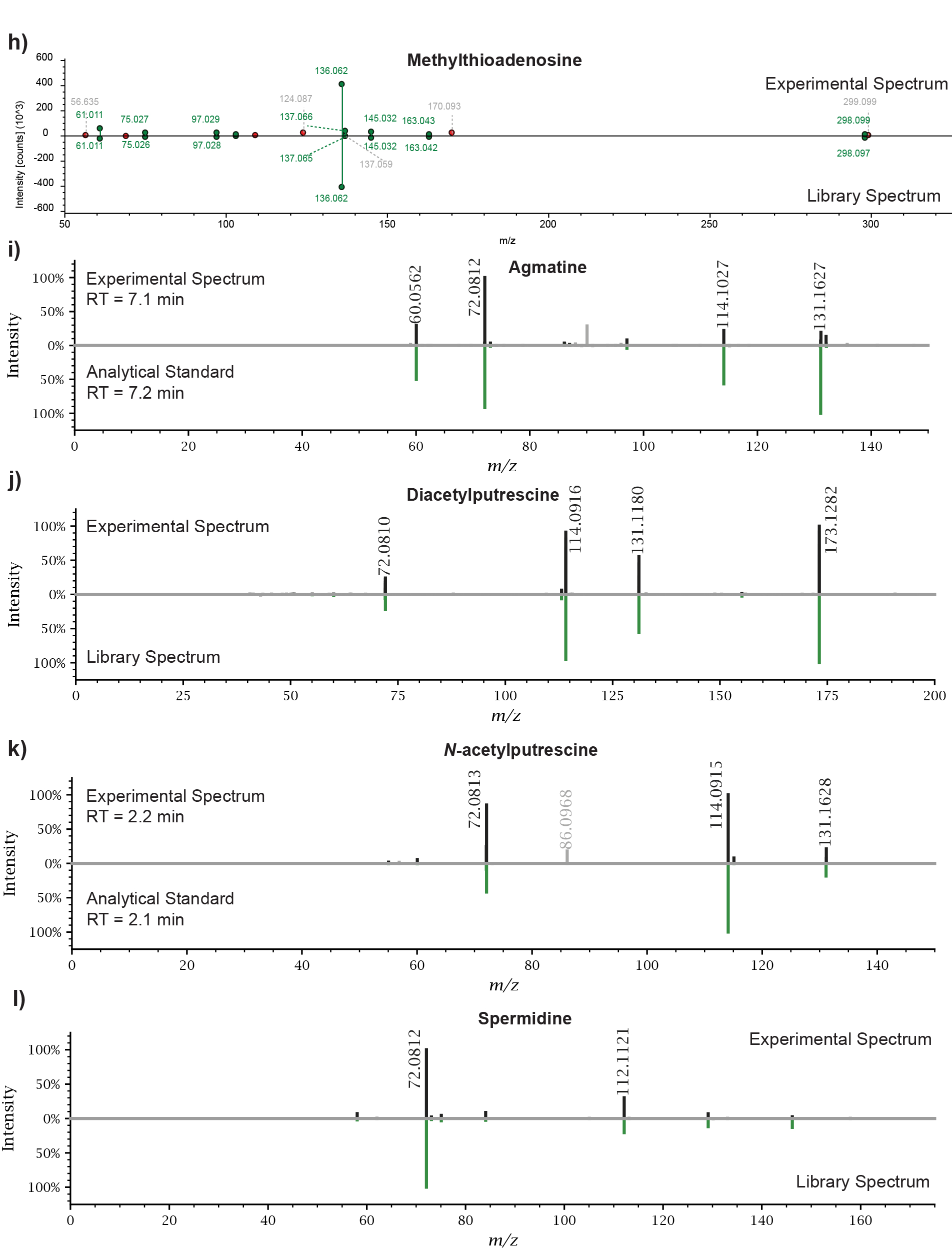


**Supplementary Figure 4**. **MS^2^ mirror plots of polyamine pathway metabolites.** A representative mirror plot of the experimental MS^2^ spectrum (top) *versus* library spectrum or analytical standard (bottom) is shown for each metabolite indicated.


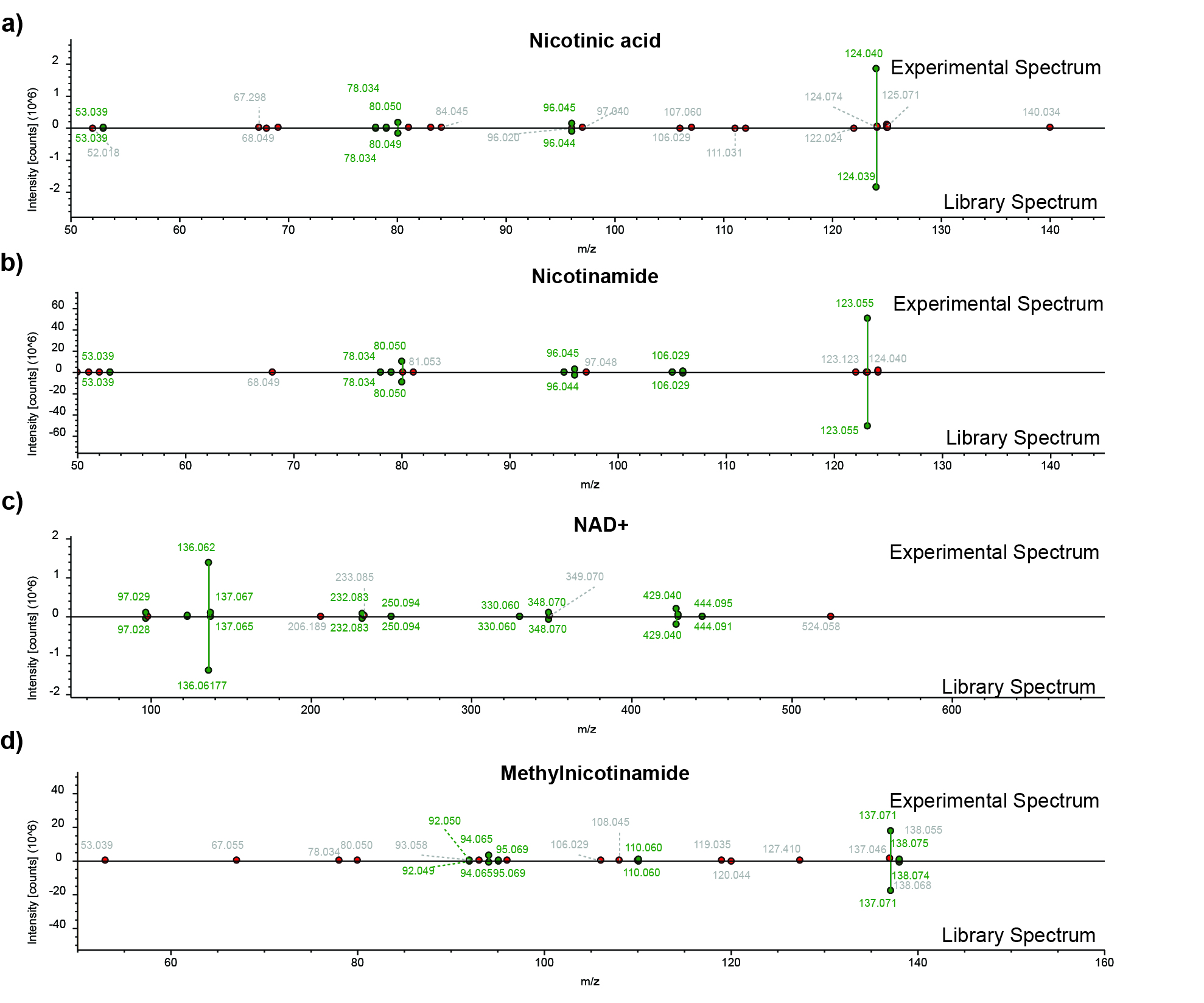
 **Supplementary Figure 5**. **MS^2^ mirror plots of NAD+ salvage pathway metabolites.** A representative mirror plot of the experimental MS^2^ spectrum (top) *versus* library spectrum or analytical standard (bottom) is shown for each metabolite indicated.


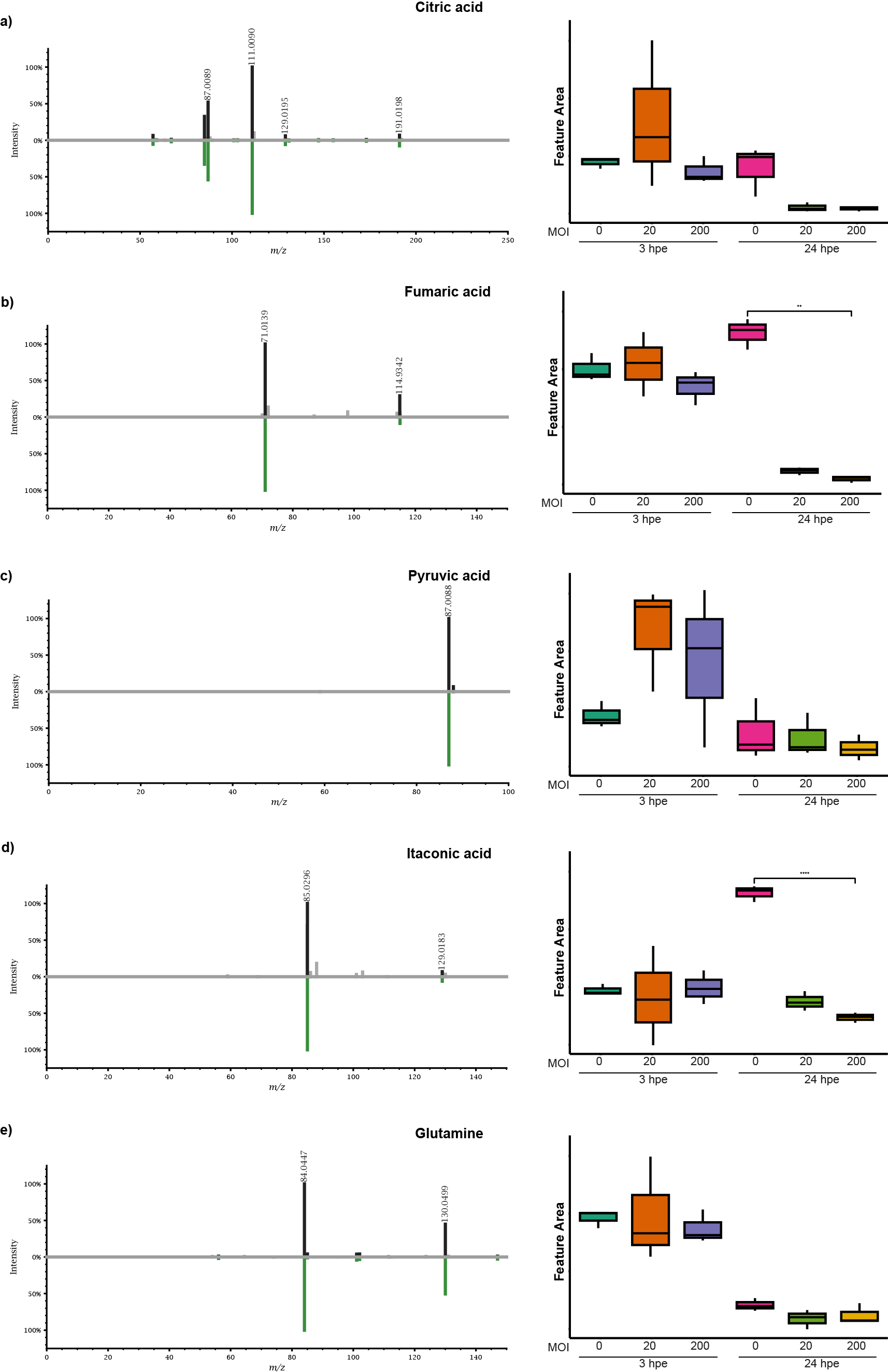


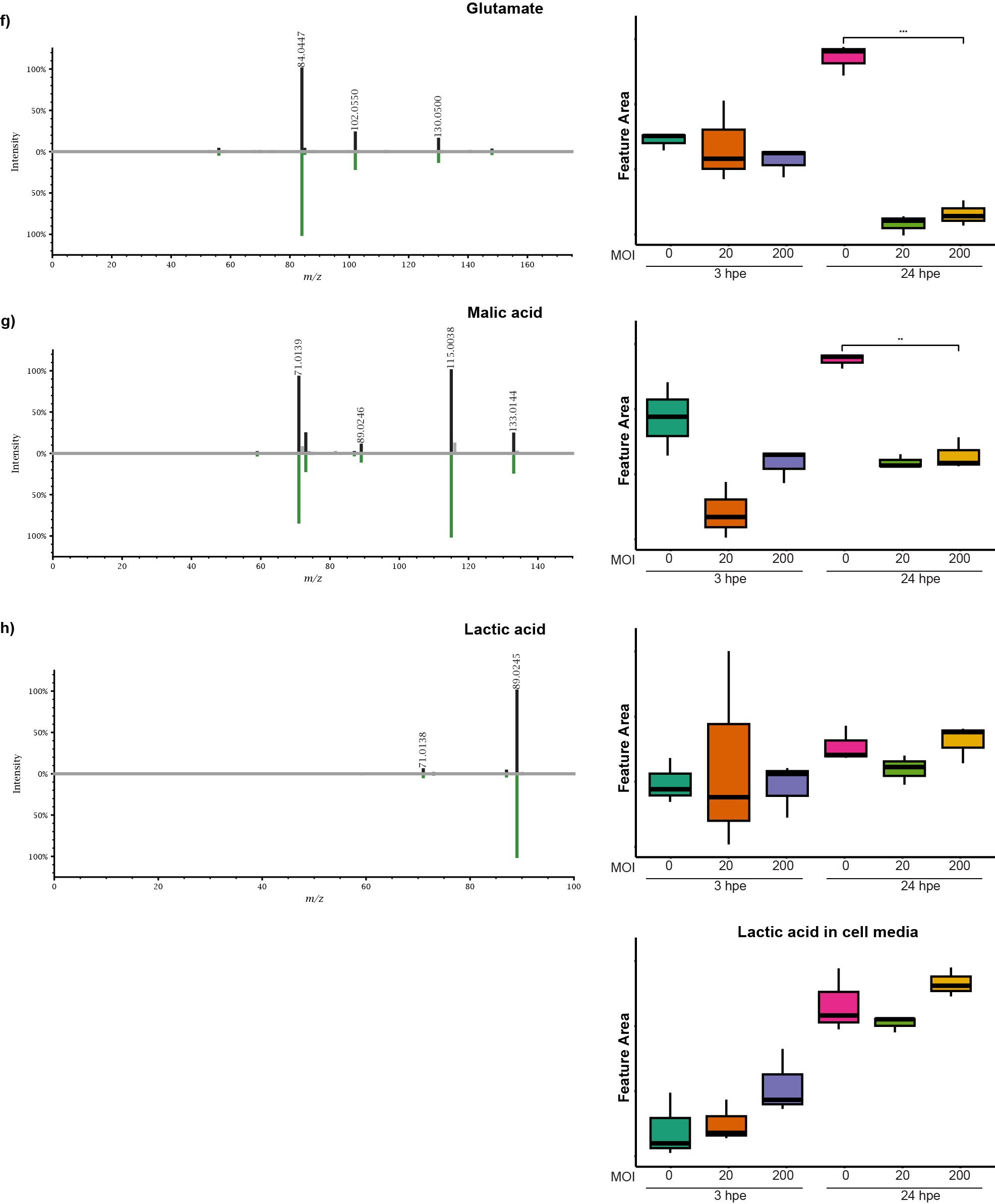


**Supplementary Figure 6. MS^2^ mirror plots and boxplots of TCA cycle metabolites*.*** For each metabolite indicated, Left: A representative mirror plot of the experimental MS^2^ spectrum (top) *versus* library spectrum or analytical standard (bottom) is shown; and Right: A boxplot of relative abundances in mock- *versus* Bt-challenged AECs is shown. In each boxplot, the box indicates the central 50% (interquartile range), the vertical lines indicate the range of the remaining data, and the horizontal line indicates the mean value for the measured abundance of the given metabolite. Asterisks indicate significant differences between the compared groups, as determined by *t* test. * refers to *p*-value < 0.05, ** refers to *p*-value < 0.01, *** refers to *p*-value < 0.001, **** refers to *p*-value < 0.0001. Note that in panel h (lactic acid), the first boxplot shows the abundances measured in each co-culture's cells (AECs and any internalized Bt), as for the other panels (a-g); whereas the second boxplot shows the abundances measured in each co-culture's conditioned medium.


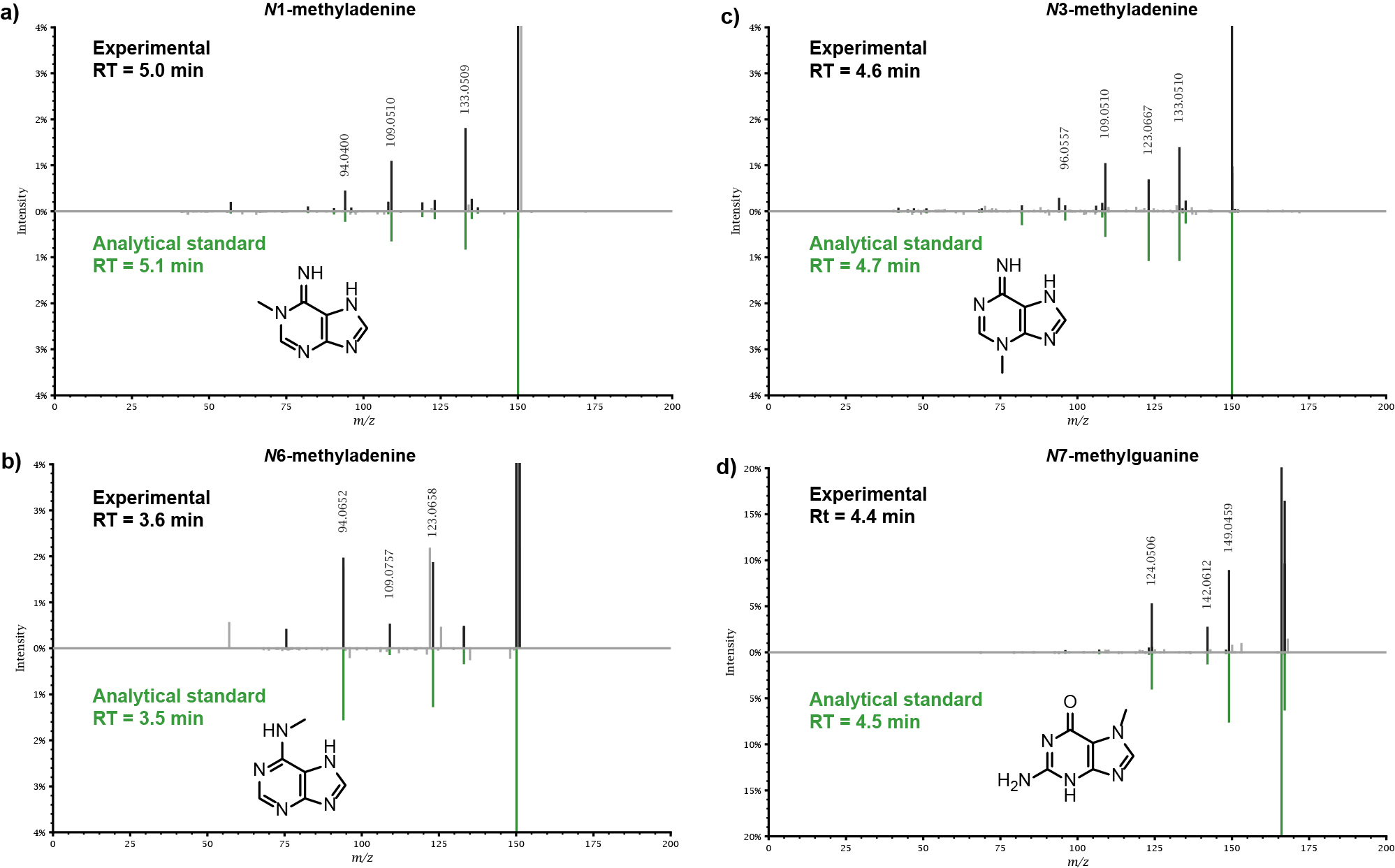


**Supplementary Figure 7*.* MS^2^ mirror plots of methylated nitrogenous base metabolites.** A representative mirror plot of the experimental MS^2^ spectrum (top) *versus* library spectrum or analytical standard (bottom) is shown for each metabolite indicated.


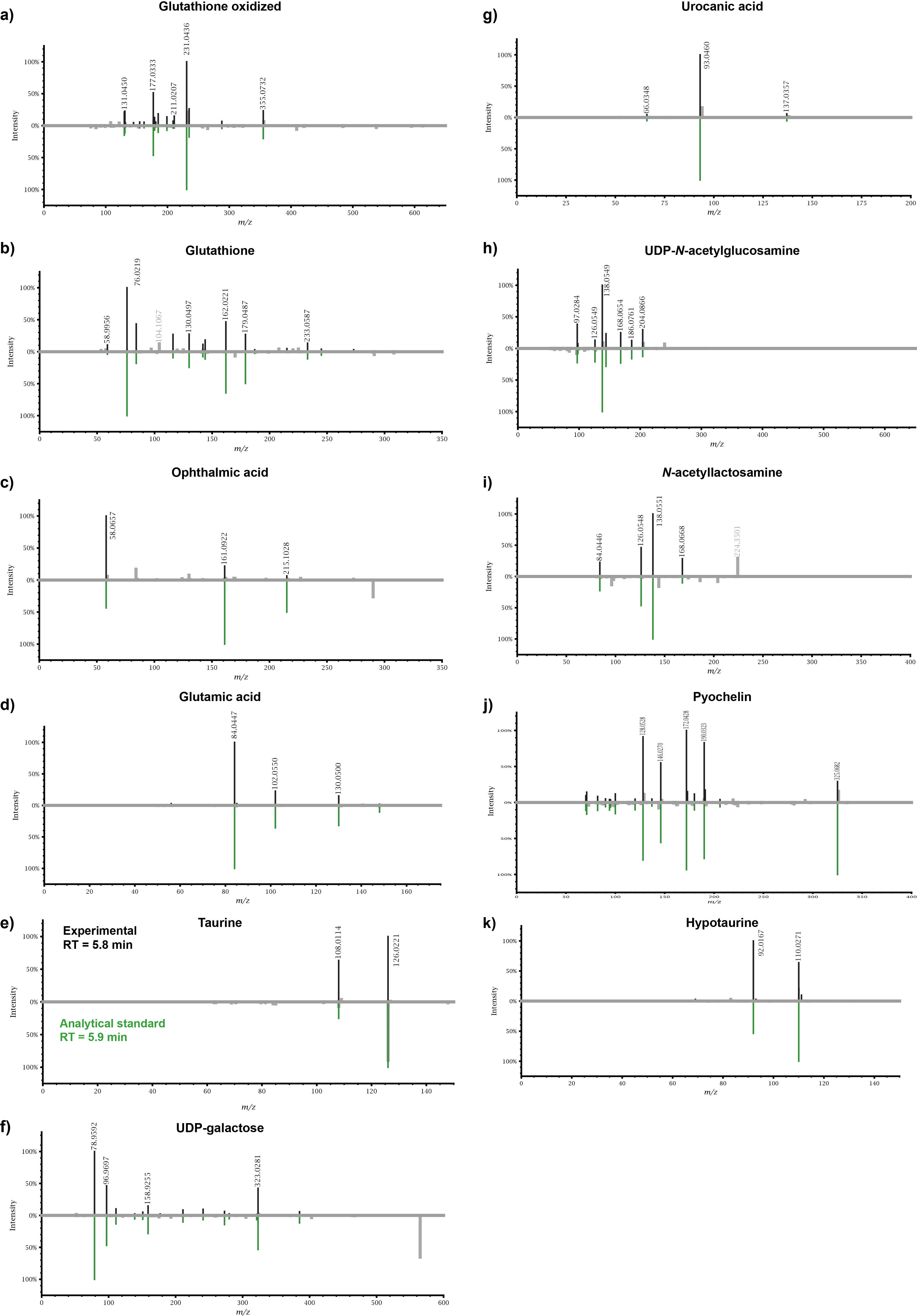


**Supplementary Figure 8. MS^2^ mirror plots of additional metabolites.** A representative mirror plot of the experimental MS^2^ spectrum (top) *versus* library spectrum or analytical standard (bottom) is shown for each metabolite indicated.


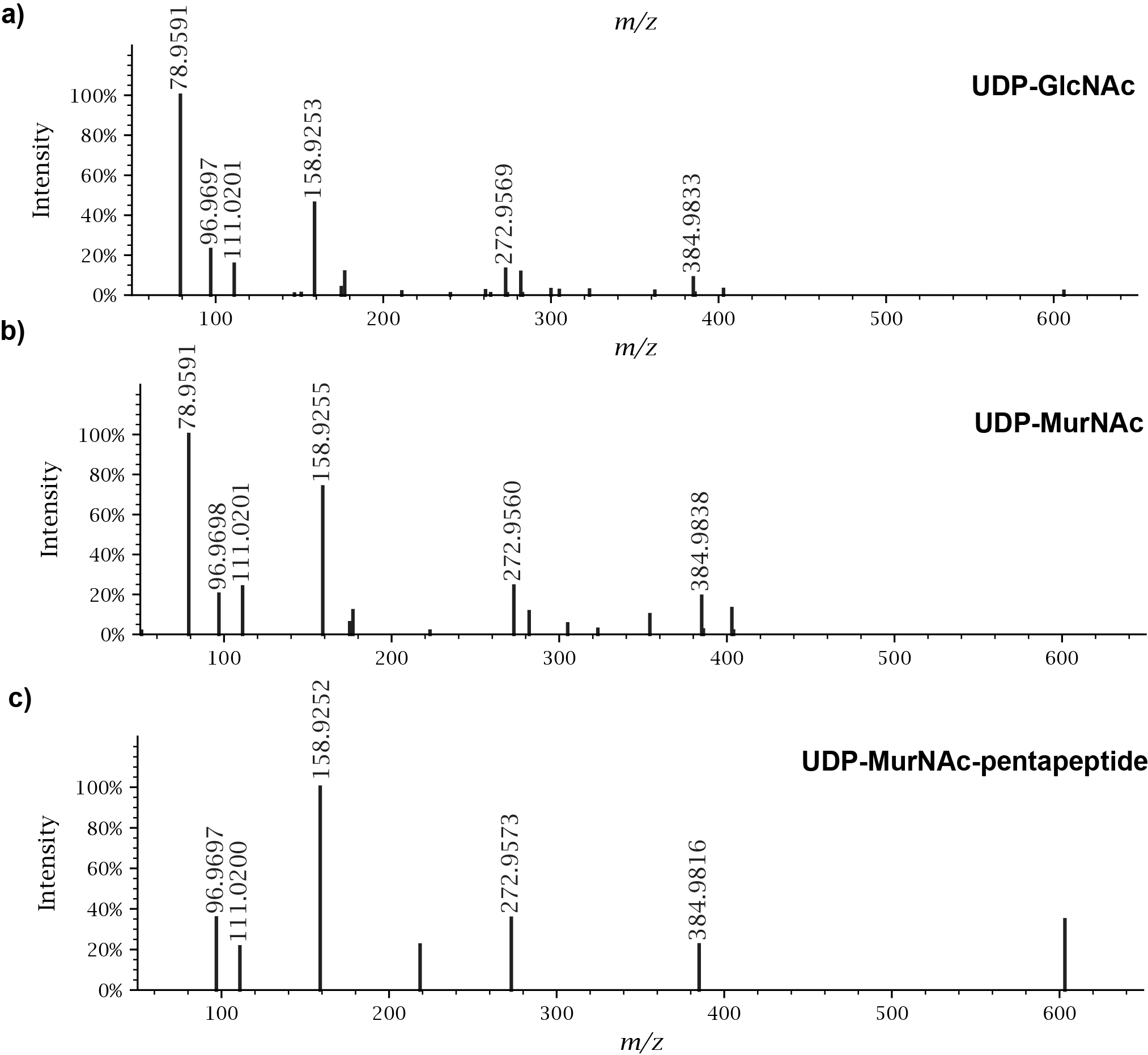
**Supplementary Figure 9. Annotation of peptidoglycan intermediates.** The experimental MS^2^ spectra of **a)** UDP-GlcNAc, **b)** UDP-MurNAc, and **c)** UDP-MurNAc-pentapeptide are shown. Shared fragments are labeled with their respective *m/z* values.

**
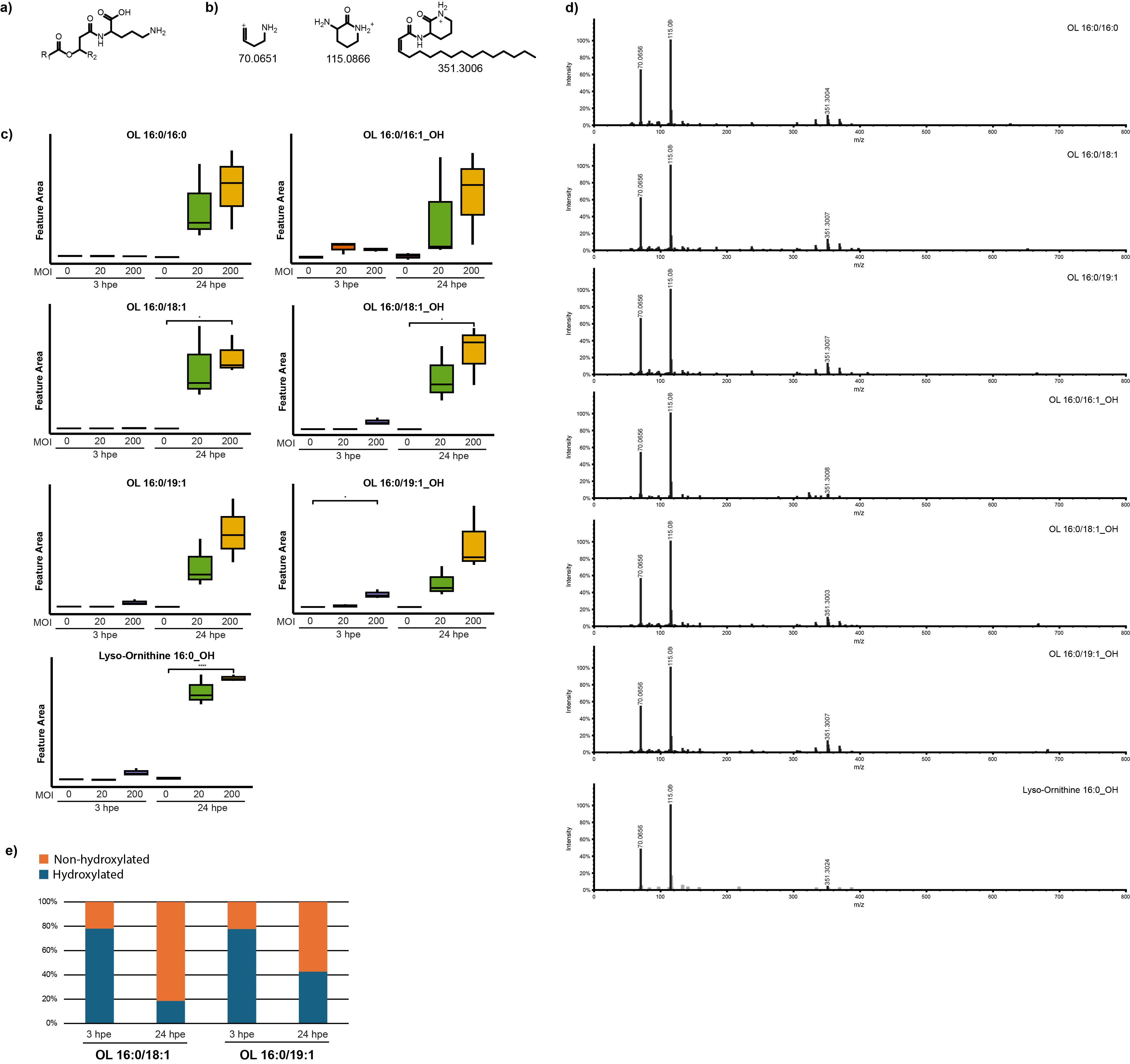
**

**Supplemental Figure 10. Statistical and spectral analyses of ornithine lipids. a)** Structure of ornithine lipid (OL). **b)** Structure of the head group fragments (70.065 and 115.087 Da) and C16:0 OL fragment (351.301 Da). **c)** Box plots of relative abundances of ornithine lipids in mock- *versus* Bt-challenged AECs*.* Each panel shows the name and detected abundance of the indicated metabolite. For each culture condition indicated, the distribution of relative abundance data is represented by a box plot in which the box indicates the central 50% (interquartile range), the vertical lines indicate the range of the remaining data, and the horizontal line indicates the mean value. Asterisks indicate significant differences between the compared groups, as determined by *t* test. * refers to *p*-value < 0.05, ** refers to *p*-value < 0.01, *** refers to *p*-value < 0.001, **** refers to *p*-value < 0.0001. **d)** MS^2^ spectra of detected ornithine lipids. **e)** Stacked bar graph showing the relative amounts of hydroxylated (blue) *versus* non-hydroxylated (orange) OL 16:0/18:1 (left) and OL 16:0/19:1 (right) detected in 200 MOI Bt:AEC co-cultures at 3 *versus* 24 hpe.

**Supplementary table 1.** Metabolite detection and annotation.

| Metabolite | PubChem CID | Polarity | M/Z | RT (min) | Annotation method |
| --- | --- | --- | --- | --- | --- |
| 5-hydroxylmethylcytosine | 70751 | + | 142.0612 | 6.3 | Literature propagation |
| Acybolin A | N/A | + | 736.309 | 1.9 | GNPS |
| Acybolin B | N/A | + | 752.303 | 2.3 | GNPS |
| Adenine | 190 | - | 134.0473 | 4.5 | MzCloud |
| Agmatine | 199 | + | 131.1292 | 8.8 | MzCloud |
| Alanine | 5950 | + | 90.0552 | 7.7 | MzCloud |
| Alanyl-alanine | 5484352 | - | 159.0778 | 8.2 | MzCloud |
| Anthranilic acid | 227 | + | 138.055 | 1.3 | NIST |
| Arginine | 6322 | + | 175.1191 | 9.1 | MzCloud |
| Bacteriohopanetetrol cyclitol ether | 101259434 | + | 708.541 | 6.2 | GNPS |
| Bactobolin A | 54676871 | + | 383.077 | 5.8 | GNPS |
| Bactobolin C | 172419372 | + | 367.083 | 5.2 | MzVault |
| Citric acid | 311 | - | 191.02 | 8.7 | MzCloud |
| Diacetylputrescine | 145677 | + | 173.1286 | 2.3 | Standard |
| fumaric acid | 444972 | - | 115.0037 | 9 | MzCloud |
| glutamic acid | 33032 | + | 148.0605 | 8.4 | MzCloud |
| Glutamine | 5961 | + | 147.0765 | 8.4 | MzCloud |
| Glutathione | 124886 | + | 308.0916 | 8.9 | NIST |
| HAQ-C9:1 db | 70272795 | + | 270.1853 | 1.1 | GNPS |
| HMAQ-C7:1db | 5459166 | + | 256.17 | 1.1 | GNPS |
| HMAQ-C9:1db | 10401590 | + | 284.201 | 1.1 | GNPS |
| HMAQ-*N*-oxide-C9:1db | 9851626 | + | 300.196 | 1.1 | GNPS |
| Hypotaurine | 107812 | + | 110.0271 | 7.9 | MzCloud |
| Indolelactic acid | 92904 | - | 204.0667 | 1.8 | MzCloud |
| Indolepyruvic acid | 803 | + | 204.0656 | 5.3 | MzCloud |
| itaconic acid | 811 | - | 129.0194 | 2.2 | MzCloud |
| Kynurenic acid | 3845 | + | 190.0499 | 5.6 | MzCloud |
| Kynurenine | 161166 | + | 209.0921 | 6.5 | MzCloud |
| Malic acid | 525 | - | 133.0143 | 6.8 | MzCloud |
| Methylnicotinamide | 457 | + | 137.0711 | 5.4 | MzCloud |
| Methylthioadenosine | 439176 | + | 298.0971 | 2.9 | MzCloud |
| *N*-(3-hydroxydecanoyl)-homoserine lactone | 137332217 | + | 290.196 | 2.2 | GNPS |
| N1-methyladenine | 135398646 | + | 150.0776 | 5.5 | Standard |
| N3-methyladenine | 135398661 | + | 150.0775 | 4.7 | Standard |
| N6-methyladenine | 67955 | + | 150.0776 | 3.5 | Standard |
| N7-methylguanine | 135398679 | + | 166.0725 | 4.9 | Standard |
| N-acetyllactosamine | 439271 | + | 383.1429 | 8.3 | MzCloud |
| N-acetylputrescine | 122356 | + | 131.118 | 5.9 | MzCloud |
| N-acetylspermine | 916 | + | 245.2339 | 9.3 | MzCloud |
| NAD+ | 15938971 | + | 664.1166 | 9.3 | MzCloud |
| Nicotinamide | 936 | + | 123.0554 | 1.8 | Standard |
| Nicotinamide riboside | 439924 | + | 255.0976 | 7.2 | MS1 and FISh scoring |
| Nicotinic acid | 938 | + | 124.0395 | 1.8 | MzCloud |
| Ophthalmic acid | 7018721 | + | 290.1349 | 8.5 | GNPS |
| Ornithine | 6262 | + | 133.0971 | 8.6 | MzCloud |
| Oxidized glutathione | 65359 | + | 613.1603 | 9.6 | MzCloud |
| Putrescine | 1045 | + | 89.1076 | 9 | Standard |
| Pyochelin | 5287441 | + | 325.068 | 1.3 | GNPS |
| pyruvic acid | 1060 | - | 87.0088 | 3.1 | MzCloud |
| S-adenosylmethionine | 34755 | + | 399.145 | 9.2 | Standard |
| Spermidine | 1102 | + | 146.1653 | 9.5 | MzCloud |
| Spermine | 1103 | + | 203.2232 | 9.9 | MzCloud |
| Taurine | 1123 | + | 126.0221 | 7.2 | Standard |
| Tryptophan | 6305 | - | 203.083 | 6.7 | MzCloud |
| UDP-Gal | 18068 | - | 565.0471 | 9.8 | NIST |
| UDP-GlcNAc | 445675 | - | 606.0754 | 9.7 | MzCloud |
| UDP-MurNAc | 11006912 | - | 678.095 | 9.6 | MS1 |
| UDP-MurNAc-pentapeptide | 5489954 | - | 595.6622 | 10.1 | MS1 |
| urocanic acid | 736715 | - | 137.0357 | 3.3 | MzCloud |
